# Mutational analysis of the *Potyviridae* transcriptional slippage site utilized for expression of the P3N-PIPO and P1N-PISPO proteins

**DOI:** 10.1093/nar/gkw441

**Published:** 2016-05-16

**Authors:** Allan Olspert, John P. Carr, Andrew E. Firth

**Affiliations:** 1 Division of Virology, Department of Pathology, University of Cambridge, Addenbrooke's Hospital, Hills Road, Cambridge CB2 0QQ, UK; 2 Department of Plant Sciences, University of Cambridge, Downing Street, Cambridge CB2 3EA, UK

## Abstract

The *Potyviridae* comprise the largest and most important family of RNA plant viruses. An essential overlapping ORF, termed *pipo*, resides in an internal region of the main polyprotein ORF. Recently, expression of *pipo* was shown to depend on programmed transcriptional slippage at a conserved GAAAAAA sequence, resulting in the insertion of an extra A into a proportion of viral transcripts, fusing the *pipo* ORF in frame with the 5′ third of the polyprotein ORF. However, the sequence features that mediate slippage have not been characterized. Using a duplicate copy of the *pipo* slip site region fused into a different genomic location where it can be freely mutated, we investigated the sequence requirements for transcriptional slippage. We find that the leading G is not strictly required, but increased flanking sequence GC content correlates with higher insertion rates. A homopolymeric hexamer is optimal for producing mainly single-nucleotide insertions. We also identify an overabundance of G to A substitutions immediately 3′-adjacent to GAAAAAA in insertion-free transcripts, which we infer to result from a ‘to-fro’ form of slippage during positive-strand synthesis. Analysis of wild-type and reverse complement sequences suggests that slippage occurs preferentially during synthesis of poly(A) and therefore occurs mainly during positive-strand synthesis.

## INTRODUCTION

Many of the most important pathogens of humans, livestock and plants are viruses with RNA genomes. Perhaps driven by their tiny genome sizes (2–32 kb), limited number of transcript species, limited access to host splicing machinery and frequent structural differences between virus transcripts and host mRNAs, RNA viruses have evolved a plethora of unusual mechanisms to express their genes. Well-known examples include internal ribosome entry site driven initiation, programmed ribosomal frameshifting and programmed stop-codon readthrough (1–2). Another such mechanism is programmed transcriptional slippage, a process in which the RNA polymerase and/or nascent strand slips in a controlled manner, leading to the addition or skipping of nucleotides in the newly synthesized RNA. As a result, different reading frames become accessible when the ‘edited’ transcripts are translated. Negative-sense single-stranded RNA viruses in the families *Paramyxoviridae* (e.g. measles and mumps viruses) and *Filoviridae* (e.g. Ebola virus) use this mechanism (3–7). In addition to viruses, transcriptional slippage is mainly known to be utilized in prokaryotes, but there are also reports from eukaryotes and chloroplasts (8–12). Recently, we and others demonstrated that programmed transcriptional slippage is widely utilized in the positive-sense RNA virus family *Potyviridae*—the largest and economically most important family of RNA plant viruses (13–16; reviewed in 17), raising the possibility that other positive-sense RNA viruses might also functionally utilize polymerase slippage for gene expression. However, the sequence requirements for efficient slippage remain poorly understood and it is not even known whether slippage occurs during positive-sense (viral genome and mRNA) or negative-sense (viral antigenome) RNA synthesis. This latter issue leads to semantic difficulties about whether the phenomenon should even be called ‘transcriptional slippage’; however since it is functionally equivalent to transcriptional slippage in other systems, we prefer the term ‘transcriptional slippage’ over the alternative ‘polymerase slippage’.

**Figure 1.**
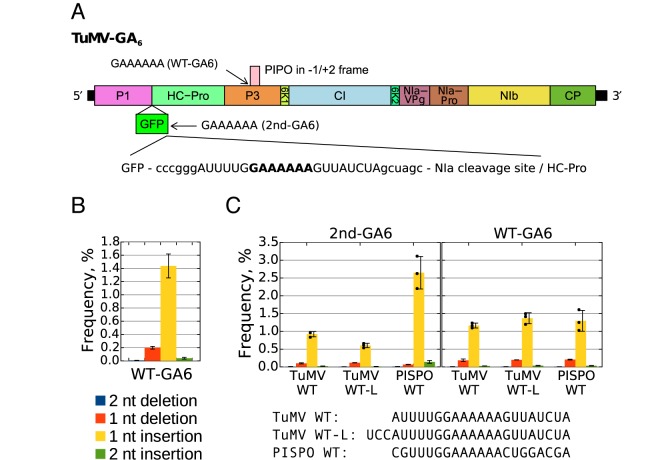
TuMV-GA6 and its utilization in slippage analysis. (**A**) Schematic of the TuMV genome showing the additional inserted 2nd-GA6 slip site. Coloured boxes indicate the polyprotein cleavage products. GFP followed by an NIa protease cleavage site is inserted between P1 and HC-Pro in the infectious clone. A small insert containing an additional GAAAAAA sequence with flanking nucleotides (2nd-GA6) was inserted between GFP and the NIa protease cleavage site as shown below the genome diagram (TuMV WT sequence shown). Regions corresponding to restriction nuclease SmaI and NheI recognition sites enabling manipulation of the 2nd-GA6 insert are indicated in lowercase. The position of the conserved GAAAAAA sequence at the 5′ end of the *pipo* ORF (WT-GA6) is indicated with an arrow. (**B**) Insertions and deletions at the WT-GA6 site. RNA was extracted at 11 d p.i. from systemically infected leaves of plants inoculated with various TuMV constructs (differences at the 2nd-GA6 site, not at WT-GA6) and subjected to targeted high-throughput sequencing. The occurrence of single or double nucleotide deletions or insertions within the slip site sequence (AAAAAA) at the WT-GA6 site was determined from 95 different biological samples (*n* = 95). Frequencies show the mean percentage of modified reads per library. Error bars indicate standard deviations. (**C**) Comparative analysis of deletions and insertions at the 2nd-GA6 and WT-GA6 sites. Plants were inoculated with viruses containing different 2nd-GA6 sites (TuMV WT, TuMV WT-L and PISPO WT) but WT TuMV sequence at the WT-GA6 site. The 2nd-GA6 sequence for each construct is shown below the graph. Systemically infected leaves were analysed as described in (A). For each construct three biological samples were tested (*n* = 3). The mean frequencies of deletions or insertions at the 2nd-GA6 site of each construct are shown in the left panel and the corresponding WT-GA6 data are shown in the right panel. Error bars indicate standard deviations; black dots mark individual samples for the single-nucleotide insertion data.

Members of the family *Potyviridae* have single-stranded positive-sense RNA genomes approximately 10 kb in length (18–19). Like other positive-sense RNA viruses, potyvirids replicate via a double-stranded RNA intermediate using a virally encoded RNA-dependent RNA polymerase, with the amount of positive-sense RNA produced during virus infection being greatly in excess of the amount of negative-sense RNA. Potyvirid genomes have a covalently linked 5′-terminal protein (VPg) and a 3′ poly(A) tail. Subgenomic transcripts are not produced. Most of the viral proteins are encoded in a single long open reading frame (ORF) that is translated as a polyprotein and cleaved to produce the mature virus proteins. However, all potyviruses contain an additional coding ORF, termed *pipo*, that overlaps the P3-encoding region of the polyprotein ORF in the −1/+2 reading frame (20). Members of the *Sweet potato feathery mottle virus* (SPFMV) subgroup contain yet another ORF, termed *pispo*, that overlaps the P1-encoding region in the −1/+2 frame (14–15,21). Recently, we and others demonstrated that both PIPO and PISPO are not expressed as independent molecules, but as fusions to the N-termini of their corresponding polyprotein products, i.e. P3N-PIPO and P1N-PISPO (20,13–16,22–24). This fusion is facilitated by transcriptional slippage during RNA synthesis. Both the *pipo* and *pispo* overlapping ORFs start at highly conserved GAAAAAA sequences that permit the viral polymerase (protein NIb, Figure 1A) to stutter and add an extra A nucleotide to a proportion of transcripts, making *pipo* (or *pispo*) accessible for translation. P3N-PIPO has been demonstrated to be required for virus cell-to-cell movement and P1N-PISPO is a suppressor of RNA silencing (14,21–25).

Insertional slippage at the *pipo* slip site occurs with an efficiency typically ranging from 0.8 to 2% (13–16). In contrast, insertional slippage at the *pispo* slip site has been reported to be 5 to 12% efficient (14,15). Differences in detection methodology, infection stage and host physiology may contribute to the reported variability. Nonetheless, it is clear that there is a degree of flexibility (or programmability) in the underlying rate of slippage. Most likely, variations in the sequence surrounding the GAAAAAA slip site modulate slippage efficiency (cf. (26)). However, more complex regulatory mechanisms (e.g. involving distal sequence elements or *trans*-acting factors) cannot be ruled out without further investigation. Bioinformatic analysis has shown that, throughout family *Potyviridae*, the GAAAAAA sequence is highly conserved at the *pipo* (and *pispo*) slip sites (13,14,20). Also, there is evidence of strong selection against six As (or six Us), but not five As (or five Us), occurring at other sites in potyviral genomes (13). This suggests the homopolymeric run as the central player in the slippage mechanism. Conservation of the leading G at the *pipo* and *pispo* slip sites implies that it also has some role in the process. To date, however, sequence analysis has given little indication as to the role of other flanking sequences in modulating slippage efficiency.

Using a duplicate copy of the *pipo* slip site region fused into a different genomic location where it can be freely mutated, we investigated the role of sequence surrounding the GAAAAAA slip site in modulating the efficiency of transcriptional slippage during potyviral infection. We reveal that the leading G is not strictly required for slippage and that slippage efficiency is modulated by the flanking nucleotides. Increased GC content upstream and/or downstream of the homopolymeric run correlates with higher insertion rates. Further, the data suggest that a homopolymeric run of 6 nt is optimal for producing mostly single-nucleotide insertions (as opposed to multiple insertions, or deletions) during slippage. We also identify an overabundance of G to A mutations immediately 3′-adjacent to the GAAAAAA sequence in insertion-free transcripts, which we infer to result from a ‘to-fro’ slippage movement during positive-strand synthesis. Analysis of wild-type (WT) and reverse complement sequences suggests that slippage occurs preferentially during synthesis of poly(A), rather than poly(U) and therefore, at the native *pipo* slip site occurs mainly during positive-strand synthesis.

## MATERIALS AND METHODS

### Viruses and plasmids

**Figure 2.**
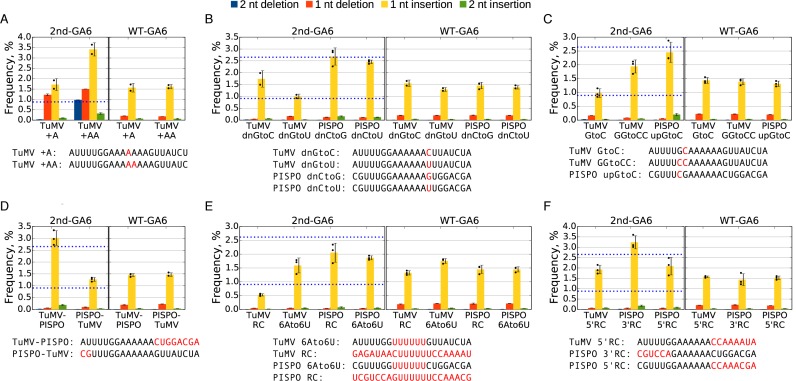
Detection of slippage events at mutated slip site sequences. (**A–F**) Plants were inoculated with various 2nd-GA6 constructs and systemically infected leaves harvested at 11 d p.i. Total RNA was extracted and subjected to targeted high-throughput sequencing. For most mutants three biological samples were used (*n* = 3), for TuMV +A, TuMV +AA, TuMV dnGtoC and TuMV dnGtoU only two biological samples were used (*n* = 2). The sequences at the 2nd-GA6 site are shown below each graph, with mutated nucleotides indicated in red. The mean frequencies of deletions or insertions at the 2nd-GA6 site of each construct are shown in the left panel and the corresponding WT-GA6 data are shown in the right panel. Error bars indicate standard deviations; black dots mark individual samples for the single-nucleotide insertion data. Reference values for TuMV WT and PISPO WT 2nd-GA6 (0.92 and 2.65%) are indicated with horizontal blue dotted lines.

**Figure 3.**
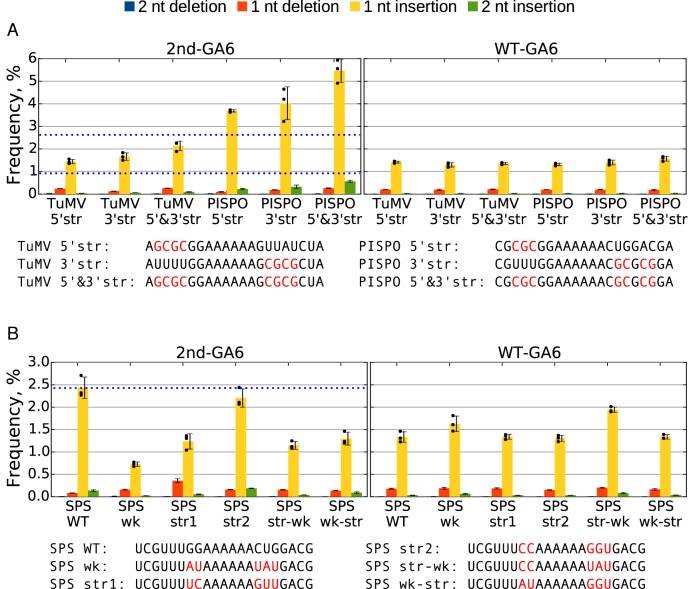
Detection of slippage events at mutated slip site sequences. (**A** and **B**) Plants were inoculated with various 2nd-GA6 constructs and systemically infected leaves harvested at 11 d p.i. Total RNA was extracted and subjected to targeted high-throughput sequencing. For each mutant three biological samples were used (*n* = 3). The sequences at the 2nd-GA6 site are shown below each graph, with mutated nucleotides indicated in red. The mean frequencies of deletions or insertions at the 2nd-GA6 site of each construct are shown in the left panel and the corresponding WT-GA6 data are shown in the right panel. Error bars indicate standard deviations; black dots mark individual samples for the single-nucleotide insertion data. Reference values for TuMV WT, PISPO WT and SPS WT 2nd-GA6 (0.92, 2.65 and 2.43%) are indicated with horizontal blue dotted lines.

GFP-tagged turnip mosaic virus (TuMV-GFP), based on isolate UK1, has GenBank accession EF028235. Reported sequence coordinates correspond to this accession. For agroinfiltration, TuMV-GFP was ligated into pGreenII as described previously (13). Mutagenesis of constructs was carried out using overlap extension polymerase chain reaction (PCR) with mutagenesis primers and standard cloning methods. The 2nd-GA6 sequence (shown in Figure 1A), together with *Sma*I and *Nhe*I restriction endonuclease sites (lowercase in Figure 1A), was inserted after *gfp* between nt 1939 and 1940. Other mutants were constructed by oligo cloning using the flanking *Sma*I and *Nhe*I sites; individual sequences without restriction sites are shown in Figures 1–3 and in Supplementary Table S1.

### Inoculation and RNA purification

*Nicotiana benthamiana* plants were grown under a 16 h photoperiod at 22°C (200 μE.m^2^.s^−1^ of photosynthetically active radiation) and 60% humidity. About 3- to 4-week old plants were inoculated by agroinfiltration as described previously (13). Briefly, *Agrobacterium tumefaciens* GV3101 containing the desired constructs was grown in LB medium at 30°C, pelleted by centrifugation at 2500 *g* at 4°C for 15 min, washed in 10 mM MgCl_2_ followed by centrifugation. Bacteria were then suspended in 0.2 mM acetosyringone in 10 mM MgCl_2_, incubated on ice for 30 min, pelleted and resuspended in the same solution. The OD600 of suspensions was adjusted to 1.0 for agroinfiltration. Samples of systemically infected leaves were collected at 11 d post-inoculation (p.i.). Leaf disks were frozen in liquid nitrogen, homogenized and total RNA was extracted as described in ref. (27).

### High-throughput sequencing

Reverse transcription was carried out on 2 μg of RNA using SuperScript III (LifeTechnologies) at 46°C for 15 min. Two primers containing the high-throughput sequencing adapter sequence and viral target sequences for sequencing the 2nd-GA6 and WT-GA6 sites, TuMV-GFP nt 1940–1963 and 3843–3859 respectively, were used together in reverse transcription. Next, excess primers were removed by 10 U of exonuclease I (NEB) at 37°C for 30 min followed by inactivation at 70°C for 15 min. PCR was performed with Q5 High-Fidelity DNA Polymerase (NEB) for 17 cycles together with high-throughput sequencing adapter primers with upstream TuMV binding sites, TuMV-GFP nt 1922–1939 and 3809–3824 for 2nd-GA6 and WT-GA6, and indexing adaptor primers matching to the first strand synthesis primers. Each biological sample was indexed separately and the PCR produced two amplicons with high-throughput adapter sites flanking viral target 2nd-GA6 and WT-GA6 sites, containing TuMV-GFP nt 1922–1963 and 3809–3859, respectively. After amplification, libraries were separated by 1× Tris-Borate-EDTA 10% polyacrylamide gel electrophoresis, target fragments were cut together from the gel and extracted. The purified libraries were quantified fluorometrically using the Qbit dsDNA HS kit (Life Technologies), normalized and sequenced using the NextSeq500 platform (Illumina). Reads were checked for quality, clipped for adapter sequence and demultiplexed based on site (2nd-GA6 and WT-GA6) using the FASTX Toolkit (Hannon lab). Reads containing Ns, too short reads, obvious contaminating reads from other libraries (errors in indexing), and reads less abundant than 1/10 000 of the most abundant read (i.e. below 0.01%) were not included in the analysis. Reads were subsequently analysed for insertions, deletions and substitutions using custom scripts mostly utilizing BioPython (28). Data previously obtained with TuMV-GFP plasmid DNA and a ΔGDD mutant, as described in ref. (13), were included as controls in the substitution profile analysis.

## RESULTS

### A 21-nt sequence containing the pipo (or pispo) slip site directs transcriptional slippage

We used a GFP-expressing infectious clone of the potyvirus TuMV (TuMV-GFP; Figure 1A). In order to study slippage and the role of nucleotides flanking the homopolymeric slip site free from restrictions imposed by the coding requirements of P3 and P3N-PIPO, a 21-nt copy (2nd-GA6) of the native *pipo* slip site and flanking sequence (WT-GA6) was inserted between the C-terminus of GFP and the NIa-protease cleavage site that defines the N-terminus of HC-Pro to make clone TuMV-GA6 (Figure 1A). In this set up, ostensibly there are no restrictions on the mutations that can be made at the 2nd-GA6 site provided no in-frame stop codons are introduced. Mutant viruses containing two slippage sites will, by independent slippage events, produce three ‘major’ RNA populations grouped by coding capacity: (i) unedited RNA expressing the polyprotein, (ii) RNA with slippage at WT-GA6 resulting in translation termination at the end of *pipo* and (iii) RNA with slippage at 2nd-GA6 (or both 2nd-GA6 and WT-GA6) resulting in translation termination shortly after the 2nd-GA6 slippage site. As the RNAs edited at either site do not produce NIb replicase *in cis*, it is our belief that they probably have similar stability and are possibly restricted from accumulation by similar replication restriction and/or decay mechanisms (13,16). The proposal that an extra slippery site will not compromise virus growth is supported by the natural occurrence of two slippage sites in SPFMV-group potyviruses at genomic positions similar to those in our mutant TuMV clone.

More than 30 clones with differently mutated 2nd-GA6 sequences were constructed (Supplementary Table S1). Each clone was introduced into three (or occasionally only two) separate plants by agroinfiltration. RNA was extracted from systemically infected leaves, the 2nd-GA6 and WT-GA6 regions were reverse transcribed, amplified and subjected to high-throughput sequencing, and the levels of insertions and/or deletions at each site for each mutant were quantified (Supplementary Table S2). Slippage rates at the unchanged WT-GA6 site were analysed for all infected plants (*n* = 95; Figure 1B). Slippage resulting in the insertion of a single A occurred in 1.44 ± 0.18% (standard deviation) of transcripts, while single-nucleotide deletions occurred in 0.20 ± 0.02% of transcripts. These values are similar, but not identical, to our previous measurements (1.9–2.1 and 0.13–0.15% respectively; *n* = 2) (13). The measured slippage rates are a combination of slippage introduced by the viral polymerase and slippage introduced during reverse transcription, PCR amplification and sequencing. Previously, we measured the latter rates for the WT-GA6 site by sequencing RNA from an agroinfiltrated ΔGDD mutant and found them to be 0.05–0.07% (single nucleotide insertions) and 0.12–0.14% (single nucleotide deletions), indicating that the bulk of insertional slippage is specific to the viral polymerase while deletional slippage may be partly or entirely explained by slippage occurring during library preparation (13).

Next we analysed slippage rates at the various mutated slip sites introduced at the 2nd-GA6 site. Initially, considering that the proportion of edited transcripts might change slightly over the course of infection (e.g. Supplementary Figure S1) or between different plants, we considered normalizing slippage rates at the mutated 2nd-GA6 site by slippage rates at the unchanged WT-GA6 site (for the same infected plant), as a means of reducing inter-biological repeat variability. However, a comparison of variability between the two sites revealed considerable scatter (Supplementary Figure S2), making such normalization futile.

Slippage rates at the 2nd-GA6 site of viruses containing the TuMV slip site (TuMV WT), a slightly longer insert (24 nt; TuMV WT-L) or the SPFMV PISPO slip site (PISPO WT) are shown in Figure 1C. For TuMV WT and TuMV WT-L, average single-nucleotide insertion rates were 0.92 ± 0.08% and 0.60 ± 0.06% respectively while, for PISPO WT, the single-nucleotide insertion rate was 2.65 ± 0.46%. Thus, a small sequence region containing the slip site is sufficient to direct slippage, and variations within this sequence region can have a substantial effect on the slippage efficiency (Supplementary Table S3 lists *t*-test values for different comparisons; *P*-values quoted in text are from two-tailed *t*-tests). Nonetheless, the slippage rates for the TuMV WT and TuMV WT-L sequences inserted at the 2nd-GA6 site differ (*P* = 0.005), and both are less than the slippage rate for the same sequence in its natural context at the WT-GA6 site (*P* = 0.002), indicating that more distant sequences likely also play a role in modulating slippage efficiency.

When the length of the homopolymeric sequence was increased by inserting an additional A (TuMV +A) or AA (TuMV +AA) (with downstream deletions to maintain reading frame) (Figure 2A), the single-nucleotide insertion rates increased to 1.72 ± 0.29% (*P* = 0.147; not statistically significant) and 3.41 ± 0.32% (*P* = 0.050), respectively. Further, the single-nucleotide deletion rates increased to 1.22 ± 0.05% and 1.50 ± 0.00% and, for TuMV +AA, 0.97% of transcripts had a 2-nt deletion. Note, however, that it is possible that the increased deletion (and insertion) rates on these longer poly(A) tracts partly reflect increased slippage during reverse transcription, PCR and sequencing. As expected, no insertional slippage was detected (<0.01%) on a run of five As (TuMV delA), or when AAAAAA was replaced with UUAAAA, AAAAUU or AUAUAU (PISPO 4A2U, PISPO 2U4A and PISPO 3(AU)) (*n* = 1; plots not shown).

### Increased GC content adjacent to the slip site promotes higher levels of slippage

Clones with the TuMV WT or PISPO WT sequence inserted at the 2nd-GA6 site (insertion rates 0.92 and 2.65%, respectively) were used as templates for further analysis of the impact of mutations flanking the slip site. The impact of the nucleotide immediately downstream of the AAAAAA (hereafter, position +7) was assessed by mutating it to either of the two other possible non-A nucleotides (Figure 2B). Mutating the downstream G to C in the TuMV context (TuMV dnGtoC) increased the insertion rate to 1.74 ± 0.35% (*P* = 0.177; not statistically significant), while changing the nucleotide to U (TuMV dnGtoU) had little apparent effect (1.00 ± 0.1%). On the other hand, changing the downstream C in the PISPO context to either G (PISPO dnCtoG) or U (PISPO dnCtoU) resulted in insertion rates (2.69 ± 0.36% and 2.46 ± 0.08%, respectively) similar to the PISPO WT sequence. Thus, single nucleotide changes next to the slip site can influence the insertion rate but effects vary depending on the surrounding nucleotide context.

Impacts of the two nucleotides immediately upstream of the AAAAAA were also assessed (Figure 2C). Changing the −1 nucleotide (following convention, our nucleotide numbering scheme omits a position 0) from G to C in the TuMV context (TuMV GtoC) did not greatly affect the insertion rate (0.98 ± 0.16%), but replacing both −2 and −1 nucleotides (TuMV GGtoCC) increased the insertion rate 2-fold (1.94 ± 0.24%; *P* = 0.011). Thus, the leading G of the highly conserved GAAAAAA *Potyviridae* slip site sequence is not essential for slippage *per se*. Similarly, when the upstream GG was replaced with CG in the PISPO context (PISPO upGtoC) the insertion rate was 2.45 ± 0.37%, similar to PISPO WT.

The role of more distant sequence elements was assessed using hybrid sequences (Figure 2D). The AAAAAA slip site flanked with TuMV upstream and PISPO downstream sequence (TuMV-PISPO) had an insertion rate of 3.02 ± 0.31% (*P* = 0.005), which is even higher than PISPO WT. Conversely, a PISPO upstream, TuMV downstream hybrid (PISPO-TuMV) had an insertion rate of 1.27 ± 0.08%, only slightly higher than TuMV WT. (It should be noted that the upstream sequences only have a 2-nt difference, 5 nt upstream from the AAAAAA.) These results suggest that the higher insertion rate of PISPO WT in comparison to TuMV WT is caused by differences in downstream sequence.

We also tested mutants where the homopolymeric sequence was changed from AAAAAA to UUUUUU (TuMV 6Ato6U and PISPO 6Ato6U) or where the whole inserted motif was replaced with its reverse complement (TuMV RC and PISPO RC) (Figure 2E). In comparison to the respective WT versions, TuMV 6Ato6U had a higher insertion rate (1.59 ± 0.27% versus 0.92 ± 0.08%; *P* = 0.042), while PISPO 6Ato6U had a lower insertion rate (1.87 ± 0.07% versus 2.65 ± 0.46%; *P* = 0.094). The reverse complement sequences had insertion rates of 0.53 ± 0.04% (TuMV RC) and 2.05 ± 0.34% (PISPO RC), both lower than the respective WT sequences (*P* = 0.004 and *P* = 0.152), though the reduction was not statistically significant for PISPO RC. In another set of mutants, only the upstream or downstream sequences were replaced with their respective reverse complements, making the sequence identical in positive- and negative-sense RNA apart from the homopolymeric hexamer (positive-sense AAAAAA, negative-sense UUUUUU) (Figure 2F). A TuMV mutant (TuMV 5′RC) in which the upstream sequence was repeated in reverse complement downstream of the AAAAAA had an insertion rate of 1.93 ± 0.21%, while a similarly generated PISPO mutant (PISPO 5′RC) had an insertion rate of 2.09 ± 0.42%. A PISPO mutant (PISPO 3′RC) with the downstream sequence repeated in reverse complement upstream of the AAAAAA had an insertion rate of 3.23 ± 0.32%.

Based on these results, we hypothesized that stretches of stronger-pairing nucleotides (i.e. G or C) upstream and/or downstream of the slip site favour higher insertion rates. This was tested with more-specific mutants (Figure 3A). Strengthening the duplex either upstream (TuMV 5′str) or downstream (TuMV 3′str) increased the insertion rate, 1.44 ± 0.1% (*P* = 0.002) and 1.66 ± 0.17% (*P* = 0.008) respectively (cf. 0.92 ± 0.08% for TuMV WT). A cumulative effect was seen when the pairing was strengthened on both sides (TuMV 5′&3′str; insertion rate 2.13 ± 0.21%; *P* = 0.005). The same trend was seen when the PISPO WT sequence was altered, with insertion rates of 3.69 ± 0.05% (*P* = 0.057), 4.02 ± 0.73% (*P* = 0.061) and 5.47 ± 0.51% (*P* = 0.002), for mutants PISPO 5′str, PISPO 3′str and PISPO 5′&3′str, respectively (cf. 2.65 ± 0.46% for PISPO WT). Strengthening the duplex downstream of the slip site seems to elevate the insertion rate slightly more than strengthening the duplex upstream.

The preceding mutations were made in the context where the WT strong-pairing GG immediately upstream of the AAAAAA was left unchanged. To test weaker-pairing nucleotides upstream and downstream of the slip site, a slightly different setup was used (Figure 3B). In order to avoid the introduction of in-frame stop codons when mutating the aforementioned GG, the PISPO WT sequence inserted at the 2nd-GA6 site was shifted 1 nt relative to the polyprotein reading frame (SPS WT). SPS WT had an insertion rate of 2.43 ± 0.24%, similar to the original PISPO WT (2.65 ± 0.46%). In this context, the duplex was weakened for (at least) three adjacent nucleotides on either side of the slip site (SPS wk). This reduced the insertion rate to 0.73 ± 0.05% (*P* = 0.005). Placing one strong-pairing nucleotide directly before and after the slip site (SPS str1) raised the insertion rate to 1.24 ± 0.17%, while placing two strong-pairing nucleotides on either side (SPS str2) further raised the insertion rate to 2.20 ± 0.2%, similar to SPS WT. A combination of two strong-pairing nucleotides upstream and two weak-pairing nucleotides downstream (SPS str-wk) had an insertion rate of 1.15 ± 0.09% while the inverse combination (SPS wk-str) had an insertion rate of 1.30 ± 0.14%. In summary, the slip site surrounded by weak-pairing nucleotides still directs mainly the introduction of insertions, but at a considerably reduced rate. The insertion rate increases when the duplex is strengthened on either side and the increase correlates with the number of strong-pairing nucleotides. The identity of individual nucleotides at each position also modulates the insertion rate.

### Transcriptional slippage preferentially occurs during synthesis of poly(A)

**Figure 4.**
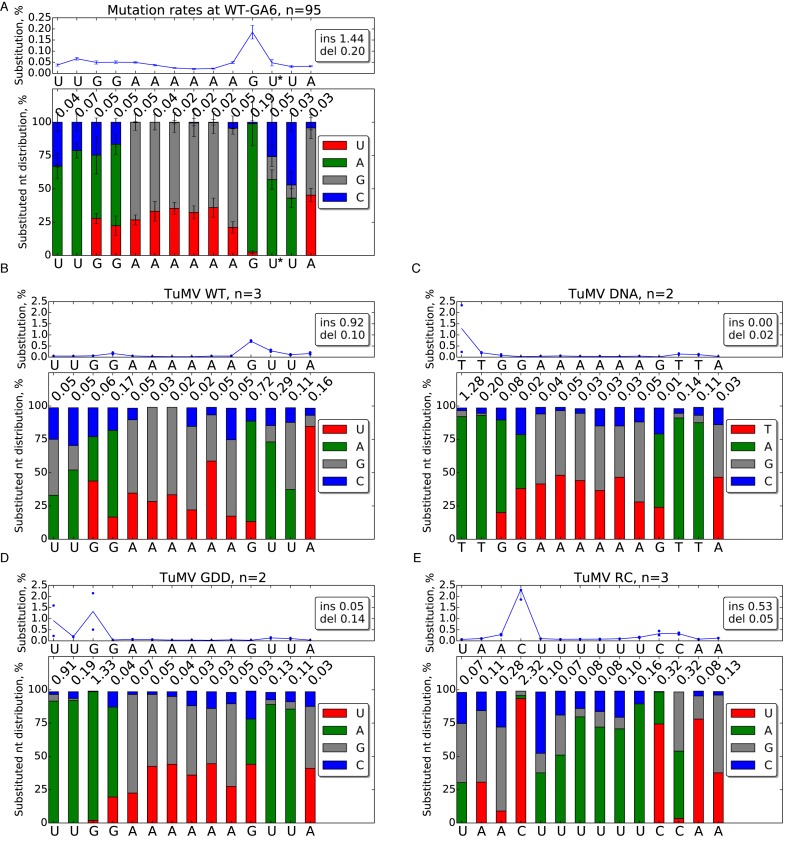
Substitution profiles at WT and mutated slip site sequences. The name and number of samples analysed is presented at the top of each subfigure. The mean frequency (blue line) of detected substitutions at each nucleotide position is shown in the upper graph of each subfigure. Error bars indicate standard deviations; for samples with two or three replicates, blue dots denote individual datapoints. The average rates of single-nucleotide insertions (ins) and deletions (del) occurring at the slip site are shown at right. The lower graph of each subfigure shows the nucleotide distribution of detected substitutions at each position. Mutations to other than the original sequence are indicated as follows: U/T—red, A—green, G—grey, C—blue. For clarity, the average total mutation rate for each position (same value as the blue line in the upper plot) is shown on top of each bar. (**A**) Mutations occurring at the WT-GA6 site across all tested constructs; *n* = 95 (at position +8 [asterisk], *n* = 94 due to the removal of a single deviantly high [by ∼2 orders of magnitude] data point). Note the different y-axis scale in this subfigure. (**B**) Mutations at the 2nd-GA6 site of construct TuMV WT. (**C**) Mutations detected using TuMV DNA (plasmid) as source, a control for mutations potentially introduced during library amplification and sequencing. (**D**) Mutations detected from *in planta* expressed TuMV RNA, a control for mutations potentially introduced during reverse transcription, library amplification and sequencing. (**E**) Mutations at the 2nd-GA6 site of construct TuMV RC.

During the course of the above analysis, it became clear that there are additional metrics that may help elucidate the mechanics of transcriptional slippage. While looking at substitution rates at the WT-GA6 site, we noticed that the G directly after AAAAAA (position +7) had a several-fold-higher substitution rate than the surrounding nucleotides (Figure 4A). Moreover, 96% of substitutions at this site were G to A. The same pattern emerged from analysis of TuMV WT sequence at the 2nd-GA6 site (76% G to A) (Figure 4B). In addition, the G directly before AAAAAA (position −1) had a much lower but nonetheless higher-than-average substitution rate, with a majority of the substitutions again being G to A. Substantially higher substitution rates were observed at the 2nd-GA6 site (0.72 ± 0.03% at position +7; *n* = 3) than at the WT-GA6 site (0.18 ± 0.02% at position +7; *n* = 95). This may partly be related to the stochastic nature of substitutions (substitutions occurring early in infection may be inherited by a larger proportion of the sequenced population) and small sample size for the WT sequence at the 2nd-GA6 site, but may also reflect purifying selection against substitutions at the WT-GA6 site (due to amino acid coding constraints in P3; Ser to Asn), whereas the 2nd-GA6 site is largely free from coding constraints. Analysis of DNA and ΔGDD controls revealed that even higher levels of substitutions can be introduced during reverse transcription, library amplification and sequencing, or potentially at other stages, but not consistently between samples nor specifically at positions −1 and +7 (Figure 4C and D).

**Figure 5.**
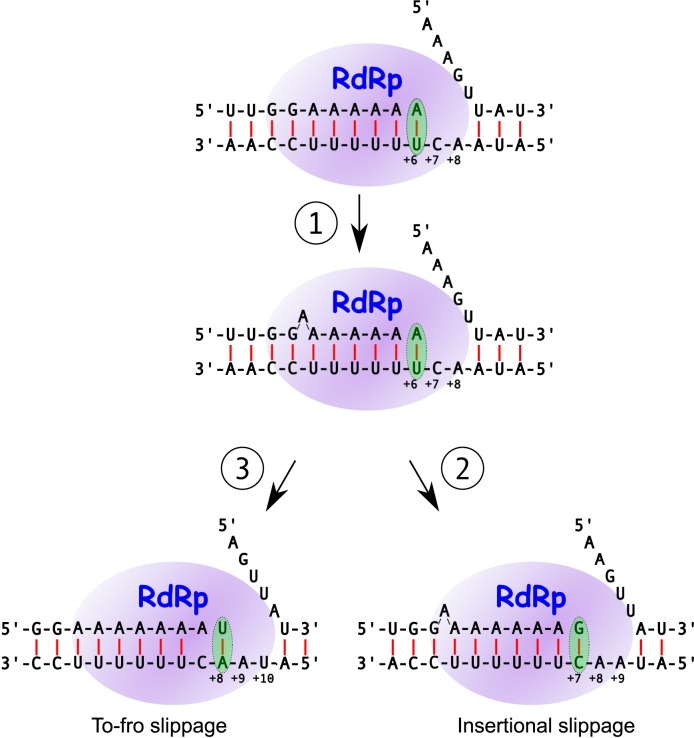
Cartoon model of insertional and to-fro slippage. At each step the polymerase is illustrated in a post-binding pre-incorporation stage. Green ellipses highlight the nucleotide entry and recognition site. Phosphodiester bonds and Watson–Crick base-pairings are indicated in black and red, respectively. (1) Following incorporation of the A templated by the +6 position, the nascent strand AAAAAA slips back 1 nt relative to the polymerase/template, resulting in a bulge nucleotide and freeing the last U of template-strand UUUUUU to template an additional A (middle). This might involve the nascent RNA slipping independently in the pre-translocation polymerase, or the polymerase and nascent RNA slipping together after translocation. Following incorporation and translocation, either (2) polymerization continues in the newly acquired register with binding of G templated by the +7 position (insertional slippage; bottom right) or (3) the nascent strand AAAAAAA slips forward 1 nt, without incorporation a second translocation occurs (possibly driven by relief of the nucleotide bulge) with A:C mispairing in the active site, and polymerization continues in the original register with binding of U templated by the +8 position (to-fro slippage; bottom left).

We hypothesize that the elevated substitution rate at position +7 results from ‘to-fro’ slippage events where the nascent RNA slips back 1 nt, allowing the polymerase to insert an extra A templated by the last U of the 3′-UUUUUU-5′ slip site antigenome sequence (as with slippage during a 1-nt insertion), but then, after translocation, the nascent RNA slips forward again and polymerization continues in the original register from position +8, thus replacing the G at +7 with an A templated from position +6 (Figure 5). Importantly, and in contrast to insertional slippage, the strandedness of to-fro slippage is easily established: G to A substitutions at position +7 must result from to-fro slippage during positive-strand synthesis. Similarly, to-fro slippage during negative-strand synthesis would explain the elevated substitution rate at position −1. The difference in substitution rates between positions −1 and +7 might reflect different efficiencies of to-fro slippage at this site during negative- and positive-strand synthesis.

Consistent with the to-fro slippage model, for TuMV RC a high level of substitutions (2.31 ± 0.47%) was observed at the −1 position and, this time, 94% of the substitutions where C to U, consistent with the C at −1 being replacing by a U templated from position +1 resulting from to-fro slippage during negative-strand synthesis (Figure 4E). Although the other UUUUUU mutants (PISPO RC, TuMV 6Ato6U and PISPO 6Ato6U) had much lower substitution rates, substitutions were again predominantly ‘to U’ at the −1 position (Supplementary Figures S4D–F). The switch in strandedness occurring when the slip site sequence was replaced with its reverse complement indicates that to-fro slippage can occur during synthesis of either positive- or negative-strand, and that strand-specificity is not dictated so much by the differing mechanics of positive- and negative-strand synthesis, but instead by the nature of the slip site sequence. In particular, to-fro slippage preferentially occurs during synthesis of poly(A), with slippage during synthesis of poly(U) being substantially less efficient (Figure 4B and E).

**Figure 6.**
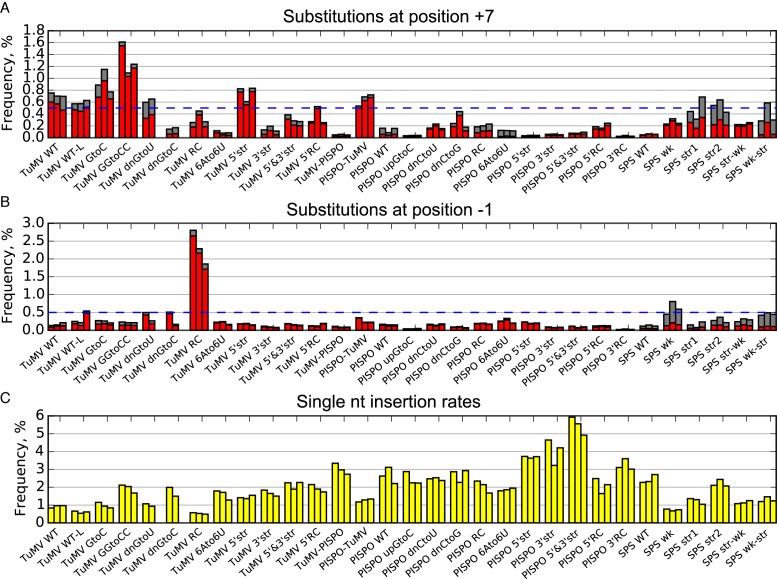
Comparison of slippage and substitution rates for different mutants. (**A** and **B**) Substitution rates at positions +7 and −1. Mutants are shown on the horizontal axis and grouped bars correspond to biological repeats (*n* = 2 or 3). Substitution rates (%) to A are in red (for the UUUUUU mutants TuMV RC, TuMV A6toU6, PISPO RC and PISPO A6toU6, substitution rates to U are in red). Substitutions to other nucleotides at the same position are plotted on top in grey. The blue dashed line corresponds to 0.5%. (**C**) Single nucleotide insertion rates (%) for the same mutants as in panels A and B.

Extending the analysis to the various mutants, we observed considerable variation in substitution rates and patterns between different sites and different mutants (Supplementary Figures S3–6). To summarize the data, we focused on mutant/position combinations with mean substitution rates greater than 0.5% (Figure 6A and B). The TuMV +A and TuMV +AA mutants were excluded due to the increased potential for contributions from the sequencing polymerases for these mutants. Eight mutants had mean substitution rates >0.5% at position +7. Six of these showed a strong preference for ‘to A’ substitutions at this site (TuMV GGtoCC—1.25% A, 0.07% U or C; TuMV GtoC—0.76% A, 0.17% U or C; TuMV 5′str—0.70% A, 0.06% U or C; TuMV WT—0.54% A, 0.17% U or C; PISPO-TuMV—0.60% A, 0.05% U or C; and TuMV WT-L—0.48% A, 0.11% U or C) while in two the preference for A over the other two possible nucleotide substitutions was decreased (TuMV dnGtoC—0.36% A, 0.27% G or C; SPS str2—0.24% A, 0.30% U or C). Notably, the six with high G to A substitution rates at position +7 all had TuMV WT sequence 3′ of the slip site. Indeed the absence of high G to A substitution rates at this position in TuMV dnGtoC or the various PISPO-based mutants, suggested that high G to A substitution rates at position +7 depend on a G at +7 and weak-pairing nucleotides at positions +8 to +11.

High substitution rates at position −1 were observed for only two mutants, TuMV RC (2.17% C to U, 0.14% C to G or A; discussed above) and SPS wk (0.62% U to A, C or G). The latter could not be explained by a to-fro slippage mechanism as most mutations were U to G rather than the expected U to A. Within the analysed region (Figure 4 and Supplementary Figures S3–6), the only other site with a mean substitution rate >0.5% was position +1 of PISPO upGtoC, where the substitution rate was 0.61 ± 0.02% and 98% of substitutions were A to G (Supplementary Figure S4C). One possible explanation for the PISPO upGtoC substitution spectrum is that it derives from ‘fro-to’ slippage (i.e. the polymerase slips forward as it would for 1-nt deletional slippage, templates a nucleotide and then slips back into the original register) during negative-strand synthesis. Since no other mutants demonstrated either ‘fro-to’ slippage, or preferential slippage during poly(U) synthesis, it would be unwise to read too much into this anomalous result without further work to definitively rule out artefacts or contamination.

## DISCUSSION

Motivated by the previous observation that the efficiency of slippage differs dramatically between two ostensibly similar slip sites (PISPO and PIPO), we decided to investigate the role of flanking nucleotides in modulating the efficiency of viral transcriptional slippage on GAAAAAA sequences. Our results indicate that slippage efficiency can be modulated by nucleotides immediately adjacent to the slip site, nucleotides within a 21-nt window encompassing the slip site, and also by more distant sequences not tested in the current analysis. Nonetheless, only mutations within the AAAAAA sequence itself completely inhibited slippage. In other words, it would appear that potyvirid species have the potential to evolve slippage rates at least in the range 0.6–6%, and, notwithstanding competing selection pressures from amino acid coding constraints, the actual slippage rates may have evolved to optimal levels for P3N-PIPO expression (low) and P1N-PISPO expression (high), while minimizing production of ‘defective’ viral transcripts.

Our study comes with a few caveats. First, we have not ruled out the possibility that the two RNA populations with an insertion either at 2nd-GA6 or WT-GA6 might have different turnover rates. Second, although we hypothesize that transcripts with insertions or deletions should not be replicated, there is some evidence to suggest that they are but at a low level (Supplementary Figure S1) so that the proportion of slippage transcripts increases slowly over the course of infection. These factors could partly explain differences between our 2nd-GA6 site TuMV and PISPO slippage rates and previously reported native-site insertion rates derived from later infection stages (13,14). Thus, the insertion rates observed at the 2nd-GA6 site should not be interpreted as absolute measurements, but can nonetheless be compared among each other.

Our results generally support the hypothesis that increased GC content upstream and/or downstream of the slip site elevates the insertion rate, although questions remain unanswered (e.g. the reasons for the roughly 3-fold difference between the ostensibly similar TuMV 5′&3′str and PISPO 5′&3′str mutants; Figure 3A). Previously we suggested that transcriptional slippage in positive-sense RNA viruses may require formation of an unpaired ‘bulge’ nucleotide (13) (see also (17)) (Figure 5), which might destabilize the duplex/replicase complex and promote replicase drop-off (cf. (29,30)). A more stable duplex upstream of the slip site might help to stabilize the post-slippage complex to counter this. In particular, the G of GAAAAAA is not required for slippage *per se* but, as its presence is highly conserved throughout family *Potyviridae*, it presumably still plays some relevant role in the process. Unfortunately, with our current approach we are not able to measure polymerase drop-off.

The role of downstream nucleotides is more difficult to understand. One possibility is that they act during negative-strand synthesis; however this would not apply if insertional slippage has a strong poly(A)-specificity like the ‘to-fro’ form of slippage (see below). It is possible that increased GC content downstream slows the replicase at the slip site by impeding strand displacement, leaving more time for slippage to occur. Cellular RNA and T7 polymerases are reported to melt only 1 bp downstream of the active site (31). On the other hand, structural data for the picornaviral polymerase—which is closely related to the potyviral polymerase (32)—indicates that the two nucleotides downstream of the templating base are unable to participate in base-pairing but sequence further downstream is duplexed (33). We propose that slippage involves the nascent strand AAAAAA slipping back 1 nt (with bulging) after incorporation of the sixth A, thus freeing the last template U for incorporation of an additional A (Figure 5). This might entail the nascent RNA slipping independently in the pre-translocation polymerase, or the polymerase and nascent RNA slipping together after translocation. At this point, the next two template nucleotides (i.e. positions +7, +8) are presumably unpaired, but nucleotides downstream (starting from position +9) may be duplexed. G/C base-pairing at position +9, potentially strengthened via stacking by G/C base-pairing at position +10, may lead to slower translocation and increased nascent RNA slippage or, alternatively, constrain a post-slippage polymerase/nascent RNA complex (at the *pispo* site these nucleotides are GG). Conversely, A/U nucleotides at these positions may result in decreased slippage. Intriguingly, these two nucleotide positions are highly conserved as U (at position +9) and to a lesser extent A (at position +10) at the *pipo* site across most potyvirus species (Figure 1 of ref. (13)). While the significance of this was not previously obvious (as conservation may simply reflect P3/PIPO amino acid constraints) it may in fact be relevant to maintaining relatively low levels of slippage at the *pipo* site.

Unexpectedly, we observed an increased rate of nucleotide substitutions at sites immediately adjacent to the slip site. The identity of these substitutions [predominantly N to A adjacent to poly(A) sites and N to U adjacent to poly(U) sites], indicated that the substitutions were largely templated by the homopolymeric sequence and occurred as a result of a ‘to-fro’ slippage. Unlike insertional or deletional slippage, the direction of synthesis is readily apparent in ‘to-fro’ slippage. High levels (>0.5%) of to-fro slippage occurred at position +7 for positive-sense AAAAAA sites and at position −1 for positive-sense UUUUUU sites, indicating that to-fro slippage was occurring during positive-strand and negative-strand synthesis, respectively. In both cases, however, high levels of slippage occurred during poly(A) synthesis, with substantially lower levels of to-fro slippage observed during poly(U) synthesis. Many RNA viruses utilize polymerase stuttering on a poly(U) template as a mechanism for maintenance of the positive-sense viral RNA 3′ poly(A) tail (34,35), and co-option of this activity for transcriptional slippage may explain the poly(A) preference. Restoration of a poly(A) tail has been reported for a potyvirus (36). Furthermore, high infectivity was maintained when there were more than five consecutive A nucleotides near the 3′ end, which might indicate the use of a stuttering/slippage mechanism. Although the results indicate that to-fro slippage can occur during synthesis of either strand (despite mechanistic differences between positive-strand and negative-strand synthesis in positive-sense RNA viruses), further studies are needed to determine whether there are fundamental differences between the two orientations in slippage propensity and/or the effect of flanking nucleotides.

To our knowledge, to-fro slippage has not been reported for other cases of transcriptional slippage and this may be related to the probable requirement for nucleotide bulging in positive-sense RNA virus transcriptional slippage, in contrast to slippage during ribonucleoprotein-templated negative-sense RNA virus transcription and cellular DNA-templated transcription, where slippage may involve complete dissociation and realignment of the nascent RNA:template strands. The unpaired bulge nucleotide may have a tendency to re-pair, driving realignment of the duplex/replicase complex back into the original register.

Although there is still no direct proof as to the strand/sequence-specificity of insertional slippage, it seems reasonable to suppose that it is closely related to the strand/sequence-specificity of to-fro slippage, since the first step of to-fro slippage is identical to insertional slippage (Figure 5). Therefore we propose that insertional slippage occurs mostly during synthesis of poly(A) (i.e. during positive-strand synthesis for WT *Potyviridae* slip sites) and only at a substantially lower level during synthesis of poly(U). This may be functionally beneficial as slippage during negative-strand synthesis would produce a viral replication complex only capable of making PIPO- or PISPO-encoding transcripts and, further, promote higher levels of additional slippage (7U template, cf. Figure 2A). The absolute levels of insertional and to-fro slippage are, however, not well correlated (Figure 5), and this may be linked to sequence contexts that drive equilibrium towards maintaining the post-slippage register or returning to the original register. Indeed only some of the tested WT and mutant sequences were subject to high levels of to-fro slippage. In particular, high levels of substitutions were observed at position +7 when the sequence 3′ of AAAAAA was TuMV WT sequence, but not when it was PISPO WT sequence. One possible contributing factor is that AAAAAA is followed by G in the TuMV WT sequence but C in the PISPO sequence. The former would lead to an A:C purine:pyrimidine nascent RNA:template mispairing following a to-fro slip, while the latter would lead to an A:G purine:purine mispairing. The purine:purine geometry might inhibit the ‘fro’ part of to-fro slippage on the PISPO sequence (leading to a corresponding increase in insertional slippage). However this cannot be the only factor (cf. PISPO dnCtoG and TuMV dnGtoU; Figure 6A).

Unlike positive-strand synthesis, negative-strand synthesis in positive-sense RNA viruses is thought to occur on a single-stranded template and not involve continuous strand displacement, but only melting of intramolecular secondary structure. Therefore it is possible that downstream sequences have differing modulatory effects on slippage during positive-strand and negative-strand synthesis. Interestingly, for the UUUUUU mutants (i.e. TuMV RC, PISPO RC, TuMV 6Ato6U and PISPO 6Ato6U), where slippage may occur predominantly during negative-strand synthesis, low insertional slippage was observed for TuMV RC only (0.53 versus 1.59–2.05%; Figure 2E). In contrast, high to-fro slippage was observed at position −1 for TuMV RC only (2.31 versus 0.19–0.26%; Figure 6B). Thus the total amount of slippage is similar, but TuMV RC (only) converts most of the slippage to the to-fro type. The reason for this may be that, of the four mutants, only TuMV RC has a pyrimidine 5′-adjacent to UUUUUU, allowing for purine:pyrimidine (rather than purine:purine) mispairing following a slip back into the original register. This supports a model (for negative-strand synthesis) where insertional slippage and to-fro slippage are in competition given an initial (sequence-determined) amount of ‘to’ slippage.

In conclusion, analysis of high-throughput sequencing data for RNA generated during infection by WT and mutant viral constructs has revealed that a significant proportion of progeny viral RNA molecules lacking any insertion at the GAAAAAA sequence instead exhibit 3′-adjacent G to A substitutions, suggesting that to-fro slippage can occur during transcription of this sequence. From this, and an analysis of reverse complement sequences, we infer that to-fro slippage is not primarily governed by any intrinsic differences between the processes of positive-sense versus antigenome-sense RNA synthesis but, rather, occurs predominantly during synthesis of poly(A). Much smaller amounts of to-fro slippage occur during synthesis of poly(U). Further work is required to verify whether insertional slippage—the form functionally utilized to express the P3N-PIPO and P1N-PISPO proteins—has the same poly(A) preference as to-fro slippage. Both to-fro and insertional slippage are affected in different ways by the identity of nucleotides flanking the shift site, with a pyrimidine 3′ adjacent to the GAAAAAA, and increased GC content upstream and/or downstream of the slip site, generally leading to higher levels of insertional slippage. These results are likely to be relevant to understanding the infidelity of the polymerases of all positive-sense RNA viruses.

## Supplementary Material

SUPPLEMENTARY DATA
