# Supplementary material for: Mutational analysis of the Potyviridae transcriptional slippage site utilized for expression of the P3N-PIPO and P1N-PISPO proteins
Source: Nucleic Acids Res. 2016 May 16;44(16):7618–29. doi: 10.1093/nar/gkw441 (PMC5027478; doi:10.1093/nar/gkw441)
Supplement: SUPPLEMENTARY DATA [file supp_44_16_7618__index.html]

Mutational analysis of the Potyviridae transcriptional slippage site utilized for expression of the P3N-PIPO and P1N-PISPO proteins — SUPPLEMENTARY DATA 

# Mutational analysis of the *Potyviridae* transcriptional slippage site utilized for expression of the P3N-PIPO and P1N-PISPO proteins

## SUPPLEMENTARY DATA

- SUPPLEMENTARY DATA
