# Supplementary material for: Mutational analysis of the Potyviridae transcriptional slippage site utilized for expression of the P3N-PIPO and P1N-PISPO proteins
Source: Nucleic Acids Res. 2016 May 16;44(16):7618–29. doi: 10.1093/nar/gkw441 (PMC5027478; doi:10.1093/nar/gkw441)
Supplement: SUPPLEMENTARY DATA [file supp_gkw441_New_Supplementary.pdf]

Mutational analysis of the *Potyviridae* transcriptional slippage site utilized for expression of the P3N-PIPO and P1N-PISPO proteins

Allan Olspert, John P. Carr, and Andrew E. Firth

Appendix

Figure S1..........1
Figure S2..........2
Figure S3-S6..........2-6
Table S1..........7
Table S2..........8
Table S3..........9

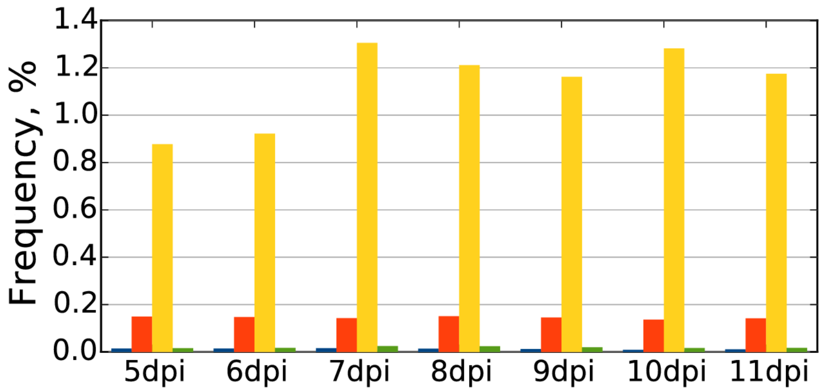

Figure S1. Slippage at the WT-GA6 site during infection progression. Samples were obtained from the upper leaves of systemically infected plants (n = 1) from 5 to 11 d p.i. (x-axis). Vertical bars represent frequencies of deletions of AA (blue), deletions of A (orange), single A insertions (yellow) and AA insertions (green).

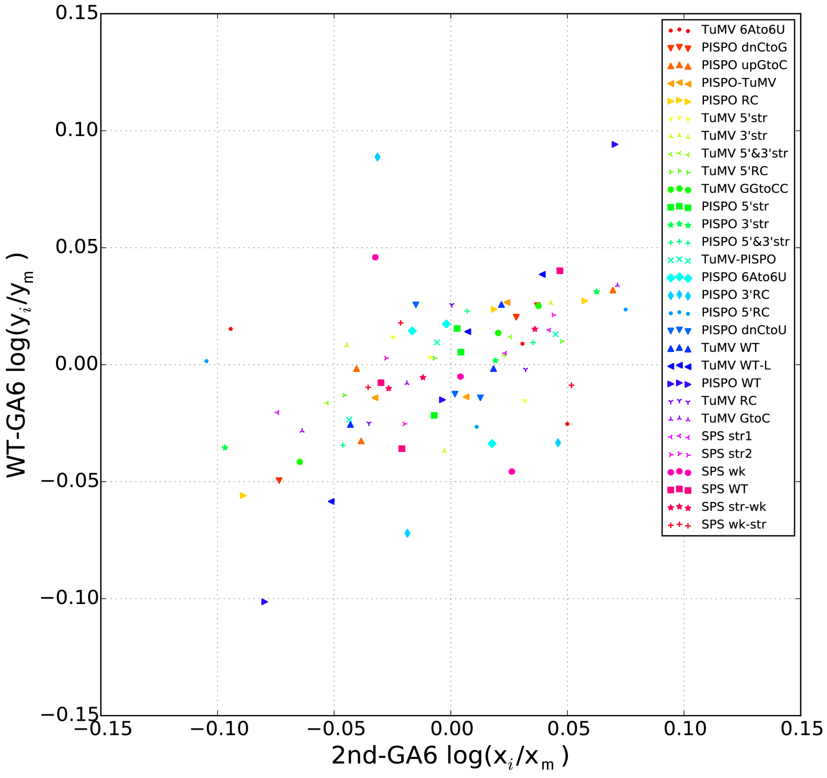

Figure S2. Comparison of variation around the mean between the 2nd-GA6 and WT-GA6 sites. For each mutant with n = 3, variation around the mean at the 2nd-GA6 site, $\log(x_i/x_m)$, was compared with variation around the mean at the WT-GA6 site, $\log(y_i/y_m)$ (where i = 1, 2, 3; x_m = mean(x_1, x_2, x_3) and y_m = mean(y_1, y_2, y_3)). Correlation between variation at the two slip sites would indicate variation in overall slippage propensity between different plants (in such cases, the WT-GA6 slippage rates might be used to normalize the 2nd-GA6 slippage rates). Instead, variation at the two slip sites was poorly correlated, indicating that variation in slippage propensity between plants is not a major contribution to overall variation.

Figures S3-S6. Substitution profiles at 2nd-GA6 slip site sequences. The name and number of samples analysed is presented at the top of each subfigure. The mean frequency (blue line) of detected substitutions at each nucleotide position is shown in the upper graph of each subfigure. Error bars indicate standard deviations; blue dots denote individual datapoints. The average rates of single-nucleotide insertions (ins) and deletions (del) occurring at the slip site are shown at right. The lower graph of each subfigure shows the nucleotide distribution of detected substitutions at each position. Mutations to other than the original sequence are indicated as follows: U – red, A – green, G – grey, C – blue. The average total mutation rate for each position (same value as the blue line in the upper plot) is shown on top of each bar.

Figure S3

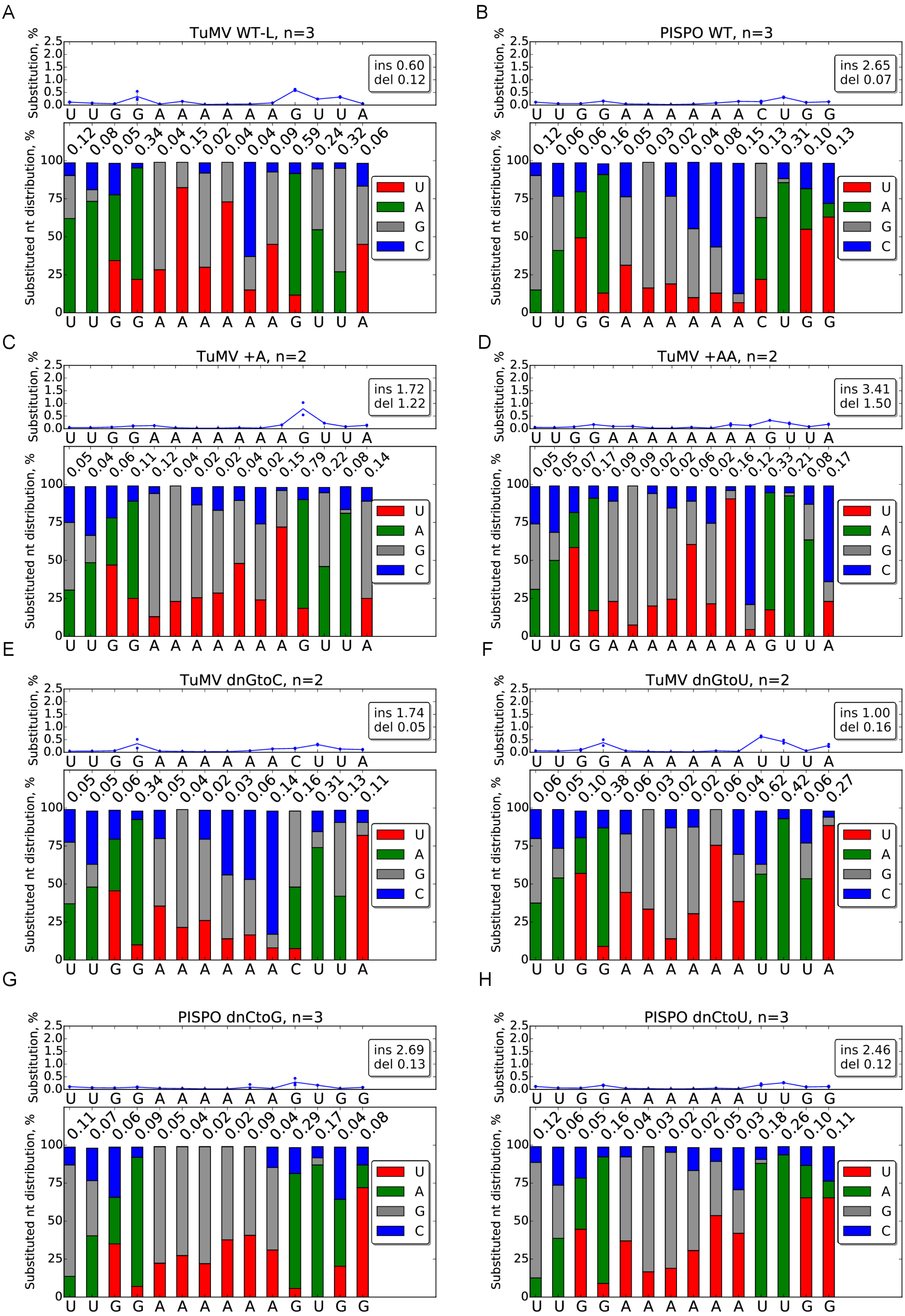
A
TuMV WT-L, n=3
ins 0.60
del 0.12
U U G G A A A A A A G U U A
0.12 0.08 0.05 0.34 0.04 0.15 0.02 0.04 0.04 0.09 0.59 0.24 0.32 0.06
B
PISPO WT, n=3
ins 2.65
del 0.07
U U G G A A A A A A C U G G
0.12 0.06 0.06 0.16 0.05 0.03 0.02 0.04 0.08 0.15 0.13 0.31 0.10 0.13
C
TuMV +A, n=2
ins 1.72
del 1.22
U U G G A A A A A A A G U U A
0.05 0.04 0.06 0.11 0.12 0.04 0.02 0.02 0.04 0.02 0.15 0.79 0.22 0.08 0.14
D
TuMV +AA, n=2
ins 3.41
del 1.50
U U G G A A A A A A A A G U U A
0.05 0.05 0.07 0.17 0.09 0.09 0.02 0.02 0.06 0.02 0.16 0.12 0.33 0.21 0.08 0.17
E
TuMV dnGtoC, n=2
ins 1.74
del 0.05
U U G G A A A A A A C U U A
0.05 0.05 0.06 0.34 0.05 0.04 0.02 0.03 0.06 0.14 0.16 0.31 0.13 0.11
F
TuMV dnGtoU, n=2
ins 1.00
del 0.16
U U G G A A A A A A U U U A
0.06 0.05 0.10 0.38 0.06 0.03 0.02 0.02 0.06 0.04 0.62 0.42 0.06 0.27
G
PISPO dnCtoG, n=3
ins 2.69
del 0.13
U U G G A A A A A A G U G G
0.11 0.07 0.06 0.09 0.05 0.04 0.02 0.02 0.09 0.04 0.29 0.17 0.04 0.08
H
PISPO dnCtoU, n=3
ins 2.46
del 0.12
U U G G A A A A A A U U G G
0.12 0.06 0.05 0.16 0.04 0.03 0.02 0.02 0.05 0.03 0.18 0.26 0.10 0.11
Substitution, %
Substituted nt distribution, %
U
A
G
C

Figure S4

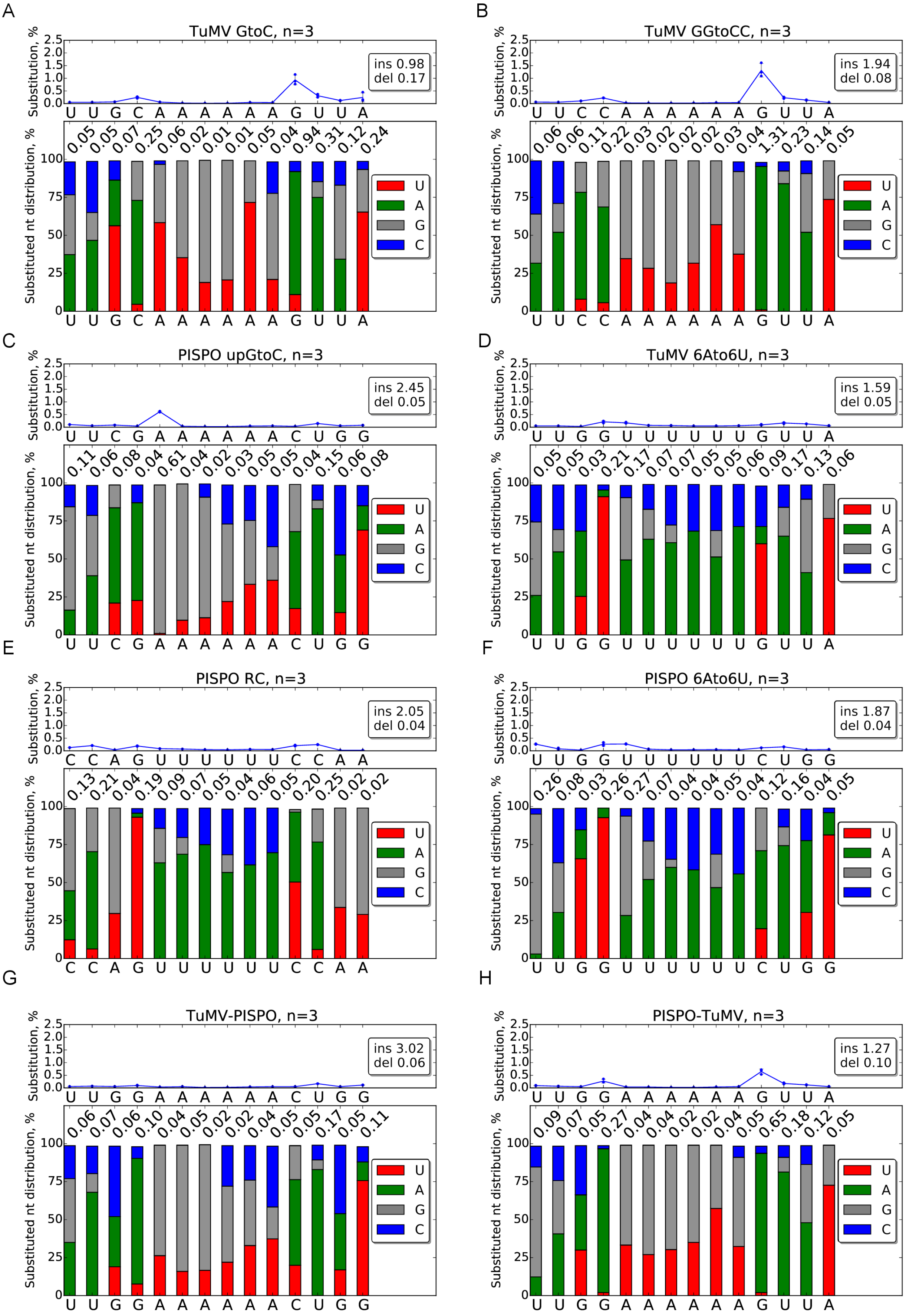
A
TuMV GtoC, n=3
ins 0.98
del 0.17
0.05 0.05 0.07 0.25 0.06 0.02 0.01 0.01 0.05 0.04 0.94 0.31 0.12 0.24
U U G C A A A A A A G U U A
B
TuMV GGtoCC, n=3
ins 1.94
del 0.08
0.06 0.06 0.11 0.22 0.03 0.02 0.02 0.02 0.03 0.04 1.31 0.23 0.14 0.05
U U C C A A A A A A G U U A
C
PISPO upGtoC, n=3
ins 2.45
del 0.05
0.11 0.06 0.08 0.04 0.61 0.04 0.02 0.03 0.05 0.05 0.04 0.15 0.06 0.08
U U C G A A A A A A C U G G
D
TuMV 6Ato6U, n=3
ins 1.59
del 0.05
0.05 0.05 0.03 0.21 0.17 0.07 0.07 0.05 0.05 0.06 0.09 0.17 0.13 0.06
U U G G U U U U U U G U U A
E
PISPO RC, n=3
ins 2.05
del 0.04
0.13 0.21 0.04 0.19 0.09 0.07 0.05 0.04 0.06 0.05 0.20 0.25 0.02 0.02
C C A G U U U U U U C C A A
F
PISPO 6Ato6U, n=3
ins 1.87
del 0.04
0.26 0.08 0.03 0.26 0.27 0.07 0.04 0.04 0.05 0.04 0.12 0.16 0.04 0.05
U U G G U U U U U U C U G G
G
TuMV-PISPO, n=3
ins 3.02
del 0.06
0.06 0.07 0.06 0.10 0.04 0.05 0.02 0.02 0.04 0.05 0.05 0.17 0.05 0.11
U U G G A A A A A A C U G G
H
PISPO-TuMV, n=3
ins 1.27
del 0.10
0.09 0.07 0.05 0.27 0.04 0.04 0.02 0.02 0.04 0.05 0.65 0.18 0.12 0.05
U U G G A A A A A A G U U A
Substitution, %
Substituted nt distribution, %
U
A
G
C

Figure S5

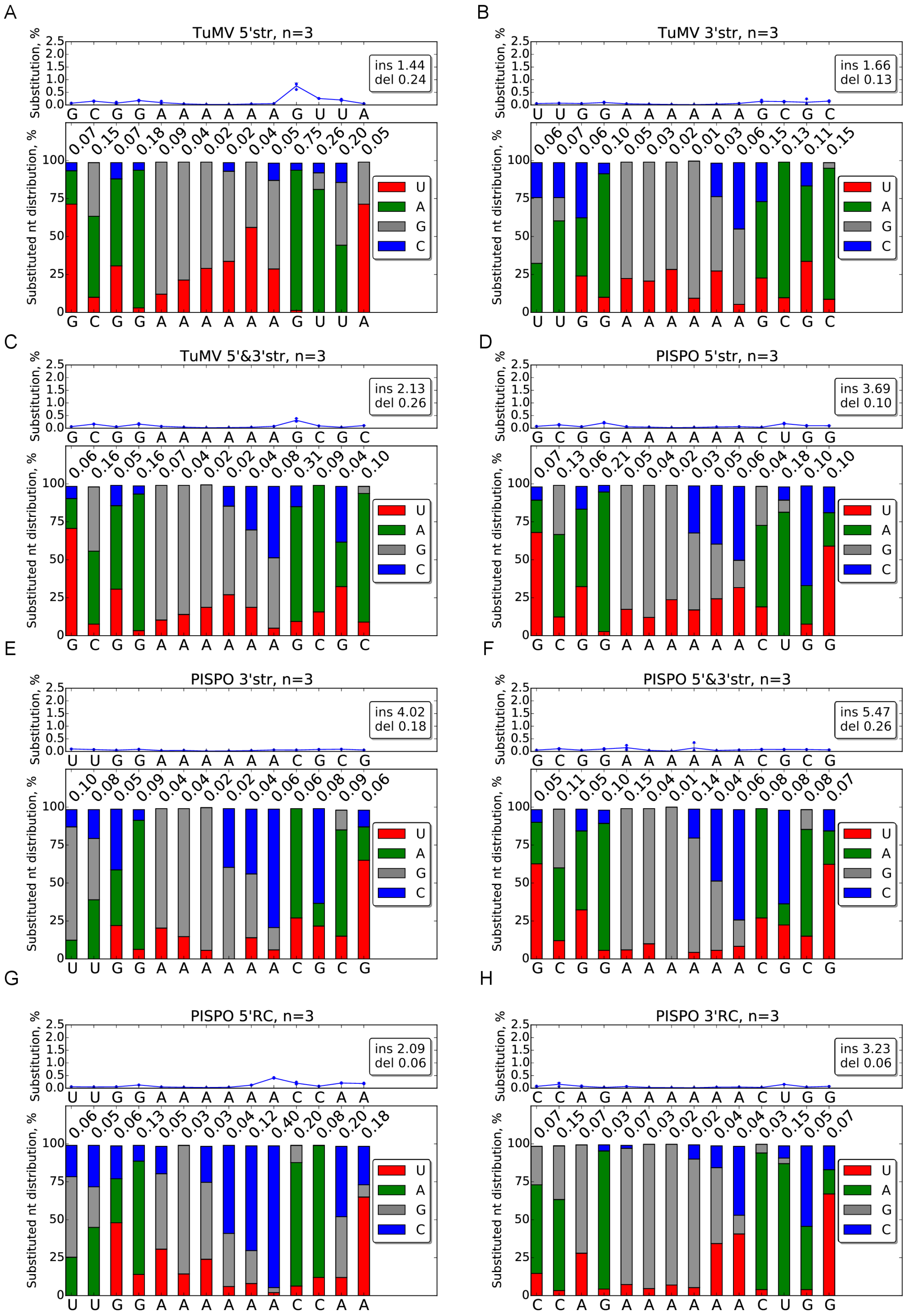

A
TuMV 5'str, n=3
ins 1.44
del 0.24
B
TuMV 3'str, n=3
ins 1.66
del 0.13
C
TuMV 5'&3'str, n=3
ins 2.13
del 0.26
D
PISPO 5'str, n=3
ins 3.69
del 0.10
E
PISPO 3'str, n=3
ins 4.02
del 0.18
F
PISPO 5'&3'str, n=3
ins 5.47
del 0.26
G
PISPO 5'RC, n=3
ins 2.09
del 0.06
H
PISPO 3'RC, n=3
ins 3.23
del 0.06
Substitution, %
Substituted nt distribution, %
U
A
G
C

Figure S6

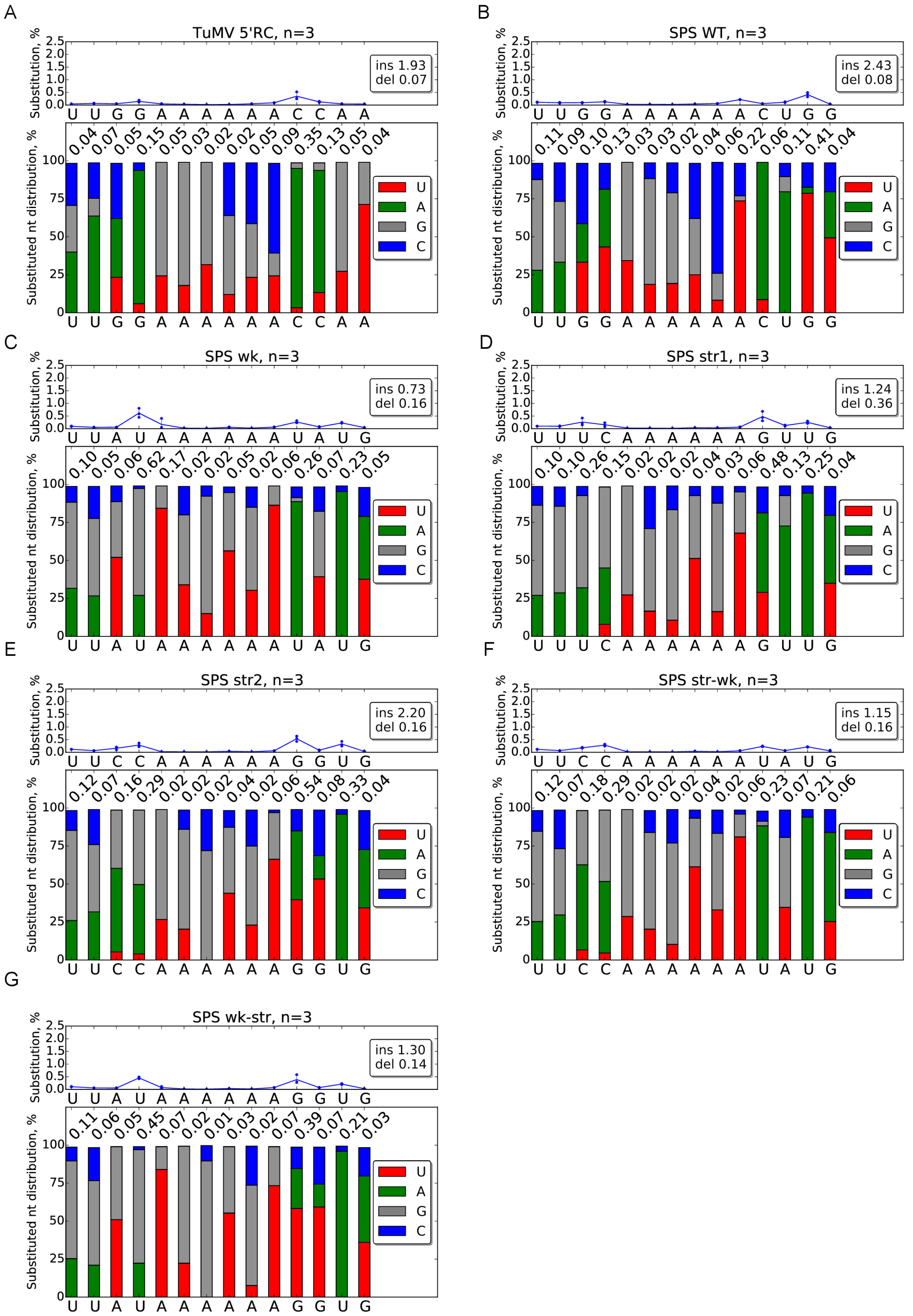
A
TuMV 5'RC, n=3
ins 1.93
del 0.07
0.04 0.07 0.05 0.15 0.05 0.03 0.02 0.02 0.05 0.09 0.35 0.13 0.05 0.04
U U G G A A A A A A C C A A
B
SPS WT, n=3
ins 2.43
del 0.08
0.11 0.09 0.10 0.13 0.03 0.03 0.02 0.04 0.06 0.22 0.06 0.11 0.41 0.04
U U G G A A A A A A C U G G
C
SPS wk, n=3
ins 0.73
del 0.16
0.10 0.05 0.06 0.62 0.17 0.02 0.02 0.05 0.02 0.06 0.26 0.07 0.23 0.05
U U A U A A A A A A U A U G
D
SPS str1, n=3
ins 1.24
del 0.36
0.10 0.10 0.26 0.15 0.02 0.02 0.02 0.04 0.03 0.06 0.48 0.13 0.25 0.04
U U U C A A A A A A G U U G
E
SPS str2, n=3
ins 2.20
del 0.16
0.12 0.07 0.16 0.29 0.02 0.02 0.02 0.04 0.02 0.06 0.54 0.08 0.33 0.04
U U C C A A A A A A G G U G
F
SPS str-wk, n=3
ins 1.15
del 0.16
0.12 0.07 0.18 0.29 0.02 0.02 0.02 0.04 0.02 0.06 0.23 0.07 0.21 0.06
U U C C A A A A A A U A U G
G
SPS wk-str, n=3
ins 1.30
del 0.14
0.11 0.06 0.05 0.45 0.07 0.02 0.01 0.03 0.02 0.07 0.39 0.07 0.21 0.03
U U A U A A A A A A G G U G
Substitution, %
Substituted nt distribution, %
U
A
G
C

Table S1. Overview of 2nd-GA6 sites.

Name	Sequence	Description
TuMV WT	AUUUUGGAAAAAAGUUAUCUA	TuMV PIPO slippage site
TuMV WT-L	UCCAUUUUGGAAAAAAGUUAUCUA	TuMV PIPO slippage site, including upstream UCC
TuMV +A	AUUUUGGAAAAAAAGUUAUCU	TuMV PIPO slippage site with 1 nt longer homopolymeric run
TuMV +AA	AUUUUGGAAAAAAAAGUUAUC	TuMV PIPO slippage site with 2 nt longer homopolymeric run
TuMV delA	AUUUUGGAAAAAGUUAUCUAC	TuMV PIPO slippage site with 1 nt shorter homopolymeric run
TuMV dnGtoC	AUUUUGGAAAAAACUUAUCUA	TuMV PIPO slippage site with downstream G (position 7) mutated to C
TuMV dnGtoU	AUUUUGGAAAAAAUUUAUCUA	TuMV PIPO slippage site with downstream G (position 7) mutated to U
TuMV GtoC	AUUUUGCAAAAAAGUUAUCUA	TuMV PIPO slippage site with upstream G (position -1) mutated to C
TuMV GGtoCC	AUUUUCCAAAAAAGUUAUCUA	TuMV PIPO slippage site with upstream GG (positions -1 and -2) mutated to CC
TuMV RC	GAGAUAACUUUUUUCCAAAAU	TuMV PIPO slippage site in reverse complement
TuMV A6toU6	AUUUUGGUUUUUUGUUAUCUA	TuMV PIPO slippage site with UUUUUU homopolymeric run
TuMV 5'str	AGCGCGGAAAAAAGUUAUCUA	TuMV PIPO slippage site with more C/G pairs upstream of homopolymeric run
TuMV 3'str	AUUUUGGAAAAAAGCGCGCUA	TuMV PIPO slippage site with more C/G pairs downstream of homopolymeric run
TuMV 5'&3'str	AGCGCGGAAAAAAGCGCGCUA	TuMV PIPO slippage site with more C/G pairs both up- and downstream of homopolymeric run
TuMV 5'RC	AUUUUGGAAAAAACCAAAAUA	TuMV PIPO slippage site which has upstream sequence in reverse complement after homopolymeric run
TuMV-PISPO	AUUUUGGAAAAAACUGGACGA	Hybrid site with TuMV PIPO upstream and SPFMV PISPO downstream sequence
PISPO-TuMV	CGUUUGGAAAAAAGUUAUCUA	Hybrid site with SPFMV PISPO upstream and TuMV PIPO downstream sequence
PISPO WT	CGUUUGGAAAAAACUGGACGA	SPFMV PISPO site
PISPO dnCtoG	CGUUUGGAAAAAAGUGGACGA	SPFMV PISPO slippage site with downstream C (position 7) mutated to G
PISPO dnC2U	CGUUUGGAAAAAAUUGGACGA	SPFMV PISPO slippage site with downstream C (position 7) mutated to U
PISPO upGtoC	CGUUUCGAAAAAACUGGACGA	SPFMV PISPO slippage site with upstream G (position -2) mutated to C
PISPO 5'str	CGCGCGGAAAAAACUGGACGA	SPFMV PISPO site with more C/G pairs upstream of homopolymeric run
PISPO 3'str	CGUUUGGAAAAAACGCGCGGA	SPFMV PISPO site with more C/G pairs downstream of homopolymeric run
PISPO 5'&3str	CGCGCGGAAAAAACGCGCGGA	SPFMV PISPO site with more C/G pairs both up- and downstream of homopolymeric run
PISPO RC	UCGUCCAGUUUUUUCCAAACG	SPFMV PISPO site in reverse complement
PISPO A6toU6	CGUUUGGUUUUUUCUGGACGA	SPFMV PISPO site with UUUUUU homopolymeric run
PISPO 3'RC	CGUCCAGAAAAAACUGGACGA	SPFMV PISPO slippage site which has downstream sequence in reverse complement before homopolymeric run
PISPO 5'RC	CGUUUGGAAAAAACCAAACGA	SPFMV PISPO slippage site which has upstream sequence in reverse complement after homopolymeric run
PISPO A/U	CGUUUGGAAAAUUCUGGACGA	SPFMV PISPO site with AAAAUU homopolymeric run
PISPO U/A	CGUUUGGUUAAAACUGGACGA	SPFMV PISPO site with UUAAAA homopolymeric run
PISPO (AU)	CGUUUGGAUAUAUCUGGACGA	SPFMV PISPO site with AUAUAU homopolymeric run
SPS WT	UCGUUUGGAAAAAACUGGACG	SPFMV PISPO site, different framing from PISPO WT
SPS wk	UCGUUUAUAAAAAAUAUGACG	SPFMV PISPO site with A/U rich up- and downstream sequence
SPS str1	UCGUUUUCAAAAAAGUUGACG	SPFMV PISPO site with one C/G nt up- and downstream of homopolymeric run
SPS str2	UCGUUUCCAAAAAAGGUGACG	SPFMV PISPO site with two C/G nt up- and downstream of homopolymeric run
SPS str-wk	UCGUUUCCAAAAAAUAUGACG	SPFMV PISPO site with two CC nt upstream and A/U rich downstream of homopolymeric run
SPS wk-str	UCGUUUAUAAAAAAGGUGACG	SPFMV PISPO site with A/U nt rich upstream and two GG nt downstream of homopolymeric run

Table S2. Mutations detected at 2nd-GA6 and WT-GA6 sites. For each biological sample (row) the frequencies (%) of A deletion(s) and A insertion(s) (or deletions/insertions of U in the case of the UUUUUU mutants TuMV RC, TuMV A6toU6, PISPO RC and PISPO A6toU6) at the homopolymeric run, together with substitution frequencies at the flanking −1 and +7 positions, are shown for both 2nd-GA6 and WT-GA6 sites.

Name	Sequence	2nd-GA6, %								WT-GA6, %							
		deletion		insertion		substitution at position -1		substitution at position 7		deletion		insertion		substitution at position -1		substitution at position 7	
		2nt	1nt	1nt	2nt	to A*	to N	to A*	to N	2nt	1nt	1nt	2nt	to A*	to N	to A*	to N
TuMV 6Ato6U	AUUUUGGUUUUUUGUUAUC	0.00	0.05	1.79	0.06	0.21	0.02	0.08	0.03	0.01	0.23	1.65	0.05	0.04	0.02	0.16	0.00
TuMV 6Ato6U	AUUUUGGUUUUUUGUUAUC	0.00	0.05	1.71	0.06	0.23	0.02	0.05	0.03	0.01	0.20	1.79	0.06	0.03	0.02	0.15	0.00
TuMV 6Ato6U	AUUUUGGUUUUUUGUUAUC	0.00	0.05	1.28	0.04	0.15	0.02	0.04	0.04	0.01	0.21	1.82	0.04	0.04	0.02	0.18	0.01
PISPO dnCtoG	CGUUUGGAAAAAAGUGGAC	0.00	0.12	2.87	0.17	0.08	0.01	0.19	0.05	0.01	0.20	1.53	0.04	0.03	0.02	0.18	0.00
PISPO dnCtoG	CGUUUGGAAAAAAGUGGAC	0.01	0.13	2.27	0.13	0.09	0.01	0.38	0.06	0.01	0.22	1.30	0.03	0.03	0.02	0.16	0.01
PISPO dnCtoG	CGUUUGGAAAAAAGUGGAC	0.01	0.12	2.93	0.18	0.06	0.01	0.12	0.06	0.01	0.19	1.55	0.05	0.02	0.02	0.14	0.01
PISPO upGtoC	CGUUUCGAAAAAACUGGAC	0.01	0.05	2.88	0.25	0.03	0.01	0.02	0.02	0.01	0.19	1.42	0.03	0.03	0.02	0.18	0.01
PISPO upGtoC	CGUUUCGAAAAAACUGGAC	0.01	0.05	2.24	0.17	0.03	0.01	0.02	0.02	0.01	0.21	1.22	0.03	0.03	0.02	0.18	0.01
PISPO upGtoC	CGUUUCGAAAAAACUGGAC	0.01	0.06	2.23	0.18	0.03	0.02	0.02	0.02	0.01	0.19	1.31	0.03	0.03	0.03	0.23	0.00
PISPO-TuMV	CGUUUGGAAAAAAGUUAUC	0.00	0.09	1.17	0.03	0.34	0.01	0.50	0.04	0.01	0.24	1.42	0.03	0.04	0.02	0.19	0.01
PISPO-TuMV	CGUUUGGAAAAAAGUUAUC	0.01	0.11	1.29	0.04	0.21	0.01	0.62	0.06	0.01	0.23	1.42	0.04	0.03	0.02	0.17	0.00
PISPO-TuMV	CGUUUGGAAAAAAGUUAUC	0.01	0.10	1.34	0.03	0.21	0.01	0.68	0.04	0.01	0.22	1.56	0.04	0.04	0.02	0.18	0.00
PISPO RC	CGUCCAGUUUUUUCCAAAC	0.00	0.04	2.35	0.10	0.18	0.01	0.08	0.10	0.01	0.23	1.54	0.04	0.04	0.02	0.16	0.01
PISPO RC	CGUCCAGUUUUUUCCAAAC	0.00	0.04	2.14	0.08	0.19	0.01	0.11	0.09	0.00	0.19	1.53	0.04	0.04	0.02	0.16	0.01
PISPO RC	CGUCCAGUUUUUUCCAAAC	0.00	0.04	1.67	0.05	0.16	0.01	0.12	0.11	0.01	0.21	1.28	0.03	0.03	0.03	0.16	0.00
TuMV 5'str	AGCGCGGAAAAAAGUUAUC	0.02	0.24	1.41	0.03	0.17	0.01	0.77	0.06	0.01	0.21	1.43	0.03	0.04	0.02	0.17	0.00
TuMV 5'str	AGCGCGGAAAAAAGUUAUC	0.03	0.25	1.36	0.04	0.17	0.02	0.55	0.05	0.01	0.20	1.46	0.03	0.03	0.02	0.16	0.01
TuMV 5'str	AGCGCGGAAAAAAGUUAUC	0.03	0.25	1.55	0.05	0.14	0.02	0.78	0.06	0.01	0.20	1.37	0.03	0.03	0.02	0.16	0.01
TuMV 3'str	AUUUUGGAAAAAAGCGCGC	0.01	0.13	1.83	0.06	0.10	0.02	0.06	0.07	0.01	0.22	1.38	0.03	0.03	0.02	0.19	0.00
TuMV 3'str	AUUUUGGAAAAAAGCGCGC	0.01	0.14	1.65	0.06	0.08	0.02	0.12	0.07	0.01	0.20	1.19	0.03	0.03	0.02	0.28	0.00
TuMV 3'str	AUUUUGGAAAAAAGCGCGC	0.01	0.12	1.50	0.06	0.07	0.01	0.05	0.06	0.01	0.17	1.32	0.03	0.02	0.02	0.17	0.00
TuMV 5'&3'str	AGCGCGGAAAAAAGCGCGC	0.02	0.26	2.25	0.09	0.17	0.01	0.31	0.07	0.01	0.23	1.37	0.03	0.04	0.02	0.18	0.00
TuMV 5'&3'str	AGCGCGGAAAAAAGCGCGC	0.02	0.25	1.89	0.09	0.14	0.01	0.21	0.07	0.01	0.20	1.30	0.03	0.03	0.02	0.16	0.00
TuMV 5'&3'str	AGCGCGGAAAAAAGCGCGC	0.02	0.27	2.26	0.11	0.13	0.02	0.20	0.07	0.01	0.22	1.39	0.03	0.03	0.02	0.15	0.00
TuMV 5'RC	AUUUUGGAAAAAACCAAAA	0.01	0.06	2.15	0.08	0.11	0.01	0.25	0.02	0.01	0.21	1.61	0.04	0.03	0.01	0.21	0.01
TuMV 5'RC	AUUUUGGAAAAAACCAAAA	0.01	0.07	1.90	0.07	0.10	0.02	0.49	0.03	0.01	0.21	1.58	0.04	0.03	0.02	0.19	0.00
TuMV 5'RC	AUUUUGGAAAAAACCAAAA	0.01	0.07	1.73	0.07	0.18	0.01	0.23	0.02	0.01	0.20	1.53	0.03	0.03	0.02	0.18	0.00
TuMV GGtoCC	AUUUUCCAAAAAAGUUAUC	0.01	0.08	2.12	0.05	0.14	0.09	1.54	0.07	0.01	0.21	1.47	0.04	0.03	0.02	0.15	0.01
TuMV GGtoCC	AUUUUCCAAAAAAGUUAUC	0.01	0.08	2.04	0.05	0.15	0.07	1.03	0.06	0.01	0.23	1.43	0.04	0.04	0.01	0.23	0.00
TuMV GGtoCC	AUUUUCCAAAAAAGUUAUC	0.01	0.09	1.67	0.05	0.13	0.08	1.17	0.07	0.01	0.22	1.26	0.03	0.03	0.02	0.16	0.00
PISPO 5'str	CGCGCGGAAAAAACUGGAC	0.01	0.09	3.73	0.26	0.22	0.01	0.02	0.02	0.01	0.20	1.33	0.03	0.03	0.01	0.15	0.01
PISPO 5'str	CGCGCGGAAAAAACUGGAC	0.02	0.10	3.63	0.21	0.18	0.02	0.03	0.02	0.01	0.20	1.25	0.03	0.03	0.02	0.16	0.00
PISPO 5'str	CGCGCGGAAAAAACUGGAC	0.03	0.11	3.71	0.21	0.19	0.02	0.02	0.02	0.01	0.19	1.36	0.03	0.03	0.02	0.16	0.00
PISPO 3'str	CGUUUGGAAAAAACGCGCG	0.01	0.19	4.65	0.37	0.08	0.01	0.04	0.01	0.01	0.21	1.50	0.04	0.03	0.02	0.27	0.01
PISPO 3'str	CGUUUGGAAAAAACGCGCG	0.01	0.20	3.22	0.24	0.07	0.01	0.04	0.02	0.01	0.19	1.29	0.03	0.03	0.02	0.17	0.01
PISPO 3'str	CGUUUGGAAAAAACGCGCG	0.01	0.17	4.21	0.37	0.07	0.01	0.04	0.01	0.01	0.20	1.41	0.03	0.03	0.02	0.16	0.00
PISPO 5'&3'str	CGCGCGGAAAAAACGCGCG	0.02	0.27	5.93	0.62	0.11	0.01	0.06	0.02	0.01	0.22	1.60	0.04	0.03	0.02	0.16	0.01
PISPO 5'&3'str	CGCGCGGAAAAAACGCGCG	0.02	0.25	5.56	0.57	0.06	0.02	0.06	0.02	0.01	0.16	1.65	0.04	0.02	0.02	0.26	0.01
PISPO 5'&3'str	CGCGCGGAAAAAACGCGCG	0.02	0.27	4.91	0.52	0.08	0.02	0.06	0.03	0.01	0.18	1.45	0.03	0.03	0.02	0.18	0.01
TuMV-PISPO	AUUUUGGAAAAAACUGGAC	0.02	0.06	3.34	0.22	0.10	0.02	0.03	0.02	0.00	0.18	1.49	0.04	0.03	0.02	0.14	0.01
TuMV-PISPO	AUUUUGGAAAAAACUGGAC	0.02	0.06	2.98	0.18	0.07	0.02	0.03	0.03	0.01	0.20	1.48	0.04	0.03	0.01	0.15	0.01
TuMV-PISPO	AUUUUGGAAAAAACUGGAC	0.02	0.07	2.73	0.16	0.07	0.01	0.03	0.02	0.01	0.21	1.37	0.03	0.03	0.02	0.16	0.00
PISPO 6Ato6U	CGUUUGGUUUUUUCUGGAC	0.00	0.04	1.80	0.05	0.25	0.01	0.02	0.10	0.01	0.21	1.50	0.04	0.03	0.02	0.15	0.00
PISPO 6Ato6U	CGUUUGGUUUUUUCUGGAC	0.00	0.04	1.86	0.06	0.29	0.04	0.03	0.09	0.01	0.21	1.51	0.04	0.03	0.02	0.15	0.00
PISPO 6Ato6U	CGUUUGGUUUUUUCUGGAC	0.00	0.05	1.94	0.06	0.19	0.01	0.02	0.09	0.01	0.22	1.34	0.04	0.03	0.01	0.18	0.00
PISPO 3'RC	CGUCCAGAAAAAACUGGAC	0.00	0.05	3.10	0.19	0.02	0.00	0.02	0.00	0.01	0.24	1.22	0.03	0.03	0.02	0.17	0.00
PISPO 3'RC	CGUCCAGAAAAAACUGGAC	0.01	0.05	3.60	0.21	0.03	0.01	0.03	0.01	0.00	0.22	1.33	0.03	0.03	0.02	0.19	0.00
PISPO 3'RC	CGUCCAGAAAAAACUGGAC	0.02	0.08	3.01	0.14	0.03	0.00	0.02	0.00	0.01	0.20	1.76	0.05	0.03	0.02	0.17	0.01
PISPO 5'RC	CGUUUGGAAAAAACCAAAC	0.00	0.07	2.48	0.11	0.09	0.03	0.15	0.04	0.01	0.19	1.61	0.05	0.03	0.02	0.17	0.01
PISPO 5'RC	CGUUUGGAAAAAACCAAAC	0.00	0.06	1.64	0.07	0.10	0.03	0.13	0.03	0.01	0.18	1.53	0.04	0.04	0.02	0.16	0.01
PISPO 5'RC	CGUUUGGAAAAAACCAAAC	0.00	0.06	2.14	0.10	0.09	0.04	0.21	0.04	0.01	0.19	1.43	0.04	0.03	0.02	0.16	0.01
PISPO dnCtoU	CGUUUGGAAAAAAUUGGAC	0.00	0.12	2.47	0.13	0.15	0.02	0.15	0.02	0.01	0.20	1.34	0.04	0.03	0.02	0.16	0.01
PISPO dnCtoU	CGUUUGGAAAAAAUUGGAC	0.01	0.11	2.53	0.13	0.12	0.03	0.20	0.03	0.01	0.19	1.34	0.04	0.03	0.02	0.15	0.01
PISPO dnCtoU	CGUUUGGAAAAAAUUGGAC	0.00	0.11	2.38	0.12	0.16	0.02	0.14	0.02	0.01	0.21	1.47	0.04	0.03	0.02	0.23	0.01
TuMV WT	AUUUUGGAAAAAAGUUAUC	0.00	0.08	0.83	0.02	0.09	0.05	0.60	0.15	0.00	0.15	1.10	0.03	0.03	0.03	0.15	0.01
TuMV WT	AUUUUGGAAAAAAGUUAUC	0.01	0.11	0.96	0.02	0.11	0.04	0.57	0.13	0.01	0.21	1.16	0.03	0.03	0.02	0.16	0.01
TuMV WT	AUUUUGGAAAAAAGUUAUC	0.01	0.11	0.97	0.02	0.13	0.09	0.46	0.23	0.01	0.20	1.23	0.03	0.03	0.02	0.19	0.01
TuMV WT-L	UCCAUUUUGGAAAAAAGUUAUC	0.01	0.12	0.66	0.02	0.19	0.06	0.47	0.10	0.01	0.20	1.50	0.04	0.03	0.02	0.17	0.01
TuMV WT-L	UCCAUUUUGGAAAAAAGUUAUC	0.01	0.11	0.54	0.01	0.13	0.08	0.45	0.13	0.01	0.20	1.20	0.03	0.03	0.02	0.15	0.01
TuMV WT-L	UCCAUUUUGGAAAAAAGUUAUC	0.01	0.12	0.61	0.01	0.48	0.06	0.51	0.11	0.01	0.20	1.41	0.04	0.03	0.02	0.15	0.01
PISPO WT	CGUUUGGAAAAAACUGGAC	0.01	0.06	2.63	0.15	0.14	0.03	0.06	0.10	0.00	0.21	1.25	0.04	0.04	0.02	0.16	0.01
PISPO WT	CGUUUGGAAAAAACUGGAC	0.01	0.07	3.11	0.18	0.12	0.04	0.04	0.04	0.01	0.19	1.61	0.04	0.03	0.02	0.17	0.01
PISPO WT	CGUUUGGAAAAAACUGGAC	0.01	0.08	2.20	0.10	0.12	0.03	0.06	0.10	0.01	0.21	1.03	0.03	0.03	0.02	0.22	0.01
TuMV RC	GAGAUAACUUUUUUCCAAA	0.00	0.05	0.57	0.02	2.65	0.16	0.18	0.08	0.00	0.20	1.32	0.04	0.03	0.02	0.19	0.01
TuMV RC	GAGAUAACUUUUUUCCAAA	0.00	0.04	0.53	0.02	2.16	0.12	0.39	0.06	0.01	0.19	1.40	0.03	0.03	0.02	0.18	0.01
TuMV RC	GAGAUAACUUUUUUCCAAA	0.00	0.05	0.49	0.01	1.71	0.15	0.19	0.08	0.00	0.18	1.25	0.03	0.03	0.02	0.16	0.01
TuMV GtoC	AUUUUGCAAAAAAGUUAUC	0.03	0.17	1.15	0.04	0.17	0.10	0.68	0.21	0.01	0.23	1.55	0.03	0.04	0.02	0.15	0.01
TuMV GtoC	AUUUUGCAAAAAAGUUAUC	0.02	0.16	0.93	0.03	0.18	0.07	0.96	0.19	0.01	0.22	1.41	0.03	0.04	0.02	0.17	0.01
TuMV GtoC	AUUUUGCAAAAAAGUUAUC	0.02	0.17	0.84	0.02	0.15	0.05	0.65	0.12	0.01	0.22	1.35	0.03	0.04	0.03	0.17	0.01
SPS str1	CGUUUUCAAAAAAGUUGAC	0.01	0.41	1.36	0.06	0.06	0.10	0.26	0.18	0.00	0.20	1.39	0.03	0.03	0.02	0.15	0.01
SPS str1	CGUUUUCAAAAAAGUUGAC	0.01	0.33	1.30	0.06	0.03	0.05	0.16	0.15	0.00	0.19	1.36	0.04	0.03	0.02	0.15	0.01
SPS str1	CGUUUUCAAAAAAGUUGAC	0.01	0.33	1.04	0.05	0.09	0.15	0.34	0.34	0.00	0.17	1.28	0.03	0.03	0.03	0.19	0.01
SPS str2	CGUUUCCAAAAAAGGUGAC	0.00	0.15	2.11	0.18	0.14	0.13	0.22	0.33	0.00	0.15	1.23	0.03	0.03	0.02	0.20	0.01
SPS str2	CGUUUCCAAAAAAGGUGAC	0.00	0.16	2.44	0.19	0.15	0.22	0.30	0.34	0.00	0.15	1.37	0.04	0.02	0.05	0.28	0.01
SPS str2	CGUUUCCAAAAAAGGUGAC	0.00	0.16	2.07	0.19	0.10	0.12	0.21	0.22	0.00	0.16	1.31	0.03	0.02	0.02	0.22	0.01
SPS wk	CGUUUAUAAAAAAUAUGAC	0.01	0.16	0.77	0.03	0.13	0.32	0.21	0.02	0.01	0.21	1.47	0.08	0.03	0.02	0.18	0.01
SPS wk	CGUUUAUAAAAAAUAUGAC	0.01	0.15	0.67	0.02	0.21	0.59	0.29	0.03	0.00	0.17	1.81	0.06	0.03	0.02	0.18	0.01
SPS wk	CGUUUAUAAAAAAUAUGAC	0.02	0.17	0.73	0.02	0.16	0.43	0.21	0.03	0.01	0.19	1.61	0.05	0.03	0.02	0.15	0.01
SPS WT	CGUUUGGAAAAAACUGGAC	0.00	0.09	2.27	0.15	0.05	0.07	0.04	0.01	0.00	0.19	1.31	0.03	0.03	0.03	0.20	0.01
SPS WT	CGUUUGGAAAAAACUGGAC	0.01	0.08	2.32	0.11	0.05	0.10	0.06	0.01	0.00	0.17	1.23	0.03	0.02	0.02	0.15	0.01
SPS WT	CGUUUGGAAAAAACUGGAC	0.00	0.08	2.71	0.15	0.05	0.07	0.05	0.01	0.00	0.18	1.46	0.04	0.03	0.02	0.28	0.01
SPS str-wk	CGUUUCCAAAAAAUAUGAC	0.01	0.17	1.08	0.04	0.12	0.12	0.19	0.03	0.00	0.20	1.90	0.07	0.03	0.03	0.18	0.01
SPS str-wk	CGUUUCCAAAAAAUAUGAC	0.01	0.15	1.12	0.04	0.15	0.17	0.19	0.03	0.00	0.19	1.92	0.09	0.03	0.02	0.21	0.01
SPS str-wk	CGUUUCCAAAAAAUAUGAC	0.00	0.15	1.25	0.04	0.13	0.16	0.23	0.02	0.00	0.21	2.01	0.09	0.03	0.02	0.19	0.01
SPS wk-str	CGUUUAUAAAAAAGGUGAC	0.00	0.14	1.20	0.08	0.10	0.32	0.05	0.23	0.00	0.18	1.31	0.03	0.03	0.02	0.19	0.01
SPS wk-str	CGUUUAUAAAAAAGGUGAC	0.00	0.13	1.46	0.11	0.11	0.38	0.26	0.33	0.00	0.15	1.31	0.03	0.02	0.02	0.20	0.01
SPS wk-str	CGUUUAUAAAAAAGGUGAC	0.00	0.14	1.24	0.09	0.10	0.34	0.06	0.24	0.00	0.17	1.39	0.04	0.03	0.02	0.20	0.01
TuMV +A	AUUUUGGAAAAAAAGUUAU	0.03	1.19	1.51	0.10	0.08	0.04	0.80	0.23	0.00	0.21	1.72	0.06	0.03	0.02	0.21	0.01
TuMV +A	AUUUUGGAAAAAAAGUUAU	0.03	1.26	1.92	0.11	0.06	0.03	0.36	0.18	0.01	0.20	1.41	0.07	0.03	0.02	0.18	0.01
TuMV +AA	AUUUUGGAAAAAAAAGUUA	0.97	1.50	3.64	0.35	0.13	0.04	0.27	0.06	0.00	0.18	1.69	0.08	0.03	0.02	0.16	0.01
TuMV +AA	AUUUUGGAAAAAAAAGUUA	0.97	1.51	3.19	0.29	0.12	0.05	0.24	0.09	0.00	0.18	1.54	0.05	0.03	0.02	0.18	0.01
TuMV dnGtoC	AUUUUGGAAAAAACUUAUC	0.02	0.05	1.99	0.06	0.47	0.04	0.06	0.08	0.01	0.21	1.65	0.05	0.03	0.02	0.19	0.01
TuMV dnGtoC	AUUUUGGAAAAAACUUAUC	0.02	0.04	1.49	0.06	0.12	0.04	0.07	0.10	0.01	0.20	1.46	0.04	0.03	0.02	0.18	0.01
TuMV dnGtoU	AUUUUGGAAAAAAUUUAUC	0.01	0.17	1.07	0.02	0.43	0.07	0.33	0.27	0.01	0.20	1.35	0.04	0.03	0.02	0.17	0.01
TuMV dnGtoU	AUUUUGGAAAAAAUUUAUC	0.01	0.16	0.93	0.02	0.19	0.08	0.39	0.26	0.01	0.19	1.24	0.03	0.03	0.02	0.19	0.01

* mutation to U with TuMV A6toU6, PISPO A6toU6, TuMV RC, PISPO RC

Table S3. Student's t-test p-values for differences in single-nucleotide insertion rates at the 2nd-GA6 site, comparing TuMV WT, PISPO WT or SPS WT sequences with their derivate mutants.

T-test		TuMV WT-L	TuMV +A	TuMV +AA	TuMV dnGtoC	TuMV dnGtoU	TuMV GtoC	TuMV GGtoCC	TuMV 5'str	TuMV 3'str	TuMV 5'&3'str	TuMV 5'RC	TuMV 6Ato6U	TuMV RC	PISPO-TuMV	TuMV-PISPO
TuMV WT	One-tailed	0.003	0.073	0.025	0.088	0.228	0.315	0.006	0.001	0.004	0.003	0.004	0.021	0.002	0.003	0.003
	Two-tailed	0.005	0.147	0.050	0.177	0.457	0.631	0.011	0.002	0.008	0.005	0.008	0.042	0.004	0.006	0.005

T-test		PISPO dnCtoG	PISPO dnCtoU	PISPO upGtoC	PISPO 5'str	PISPO 3'str	PISPO 5'&3'str	PISPO 3'RC	PISPO 5'RC	PISPO 6Ato6U	PISPO RC	PISPO-TuMV	TuMV-PISPO
PISPO WT	One-tailed	0.457	0.274	0.298	0.028	0.031	0.001	0.073	0.098	0.047	0.076	0.015	0.160
	Two-tailed	0.913	0.549	0.596	0.057	0.061	0.002	0.147	0.196	0.094	0.152	0.031	0.319

T-test		SPS wk	SPS str1	SPS str2	SPS str-wk	SPS wk-str
SPS WT	One-tailed	0.003	0.002	0.140	0.003	0.002
	Two-tailed	0.005	0.003	0.280	0.006	0.004
